# Molecular Mechanisms and Regulation of Anthocyanin Biosynthesis in Rosaceae Fruits: A Review

**DOI:** 10.1002/fsn3.72269

**Published:** 2026-09-02

**Authors:** Menghua Luo, Wanjia Tang, Xiaoqing Yuan, Can Hu

**Affiliations:** ^1^ Deyang Agricultural College Deyang China; ^2^ College of Horticulture Sichuan Agricultural University Chengdu China; ^3^ Sichuan Jiaotong Polytechnic University Chengdu China

**Keywords:** anthocyanin, biosynthesis, environmental regulation, fruit color, Rosaceae, transcriptional regulation

## Abstract

Anthocyanin accumulation in Rosaceae fruits is governed by a multi‐layered regulatory network integrating transcriptional control, epigenetic modulation, environmental signaling, and metabolic flux allocation. This review presents a critical synthesis of recent advances in this field, moving beyond descriptive gene lists to examine the mechanistic underpinnings of pigment regulation. We first outline the core biosynthetic pathway and post‐synthetic modifications that ensure pigment stability. The central focus is on the MYB–bHLH–WD40 transcriptional complex and its integration with epigenetic mechanisms (DNA methylation, histone modifications, non‐coding RNAs) and post‐transcriptional regulation. We further examine how environmental cues—light, temperature, and hormones—are transduced through these molecular networks, and analyze metabolic flux control at pathway branch points. A cross‐species comparative perspective across major Rosaceae crops (*Malus*, *Pyrus*, *Fragaria*, *Prunus*) reveals conserved regulatory nodes and genus‐specific innovations. Finally, we discuss how this integrated knowledge, combined with multi‐omics and gene editing tools, is driving targeted strategies in molecular breeding and postharvest quality management. This framework provides a foundation for rational manipulation of fruit coloration and nutritional quality.

## Graphical abstract

Five‐tiered regulatory network governing anthocyanin biosynthesis in Rosaceae fruits, integrating environmental signals, epigenetic/transcriptional control, flavonoid metabolism, pigment trafficking and breeding translation. This review elaborates layered regulatory networks underlying anthocyanin production in core Rosaceae crops (*Malus*, *Pyrus*, *Fragaria*, *Prunus*). Starting with the conserved phenylpropanoid‐flavonoid pathway, we describe structural enzymes, metabolic flux partitioning, post‐synthetic pigment modification and vacuolar transport mediated by GST/MATE/ABC transporters. The central MBW ternary complex, together with auxiliary transcription factors, epigenetic remodeling and non‐coding RNA‐mediated post‐transcriptional regulation, controls pigment accumulation. Light, temperature and phytohormone signals converge to modulate MBW activity, while cross‐genus comparison uncovers conserved modules and lineage‐specific variations shaped by *MYB10* allelic divergence. Translational applications including molecular breeding, gene editing and postharvest management are summarized, and key future research directions are proposed. This integrated schematic establishes a unified framework for optimizing fruit color and nutritional value in Rosaceae.

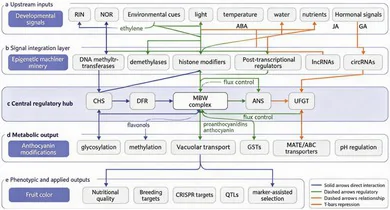

## 1 Introduction

The Rosaceae family encompasses many of the world's most economically important fruit crops, such as apple ( *Malus domestica* ), pear (*Pyrus* spp.), peach ( *Prunus persica* ), strawberry (*Fragaria* × *ananassa*), sweet cherry ( *Prunus avium* ), raspberry, and blackberry (*Rubus* spp.) (Liu et al. 2020). Fruit color is a major visual quality attribute that strongly influences marketability and consumer acceptance (Jiang et al. 2019). Vivid, uniform red, purple, or blue‐black hues are often instinctively associated with optimal ripeness, desirable flavor, and higher nutritional value. This coloration is largely attributed to the biosynthesis and tissue‐specific accumulation of anthocyanins, a prominent class of water‐soluble flavonoids (Zhang et al. 2025). Ultimately, the color phenotype of Rosaceae fruits is directly and intricately shaped by anthocyanin concentration, chemical composition, and spatiotemporal distribution patterns, which in turn significantly affect commercial grading and consumer acceptance.

Anthocyanins are ubiquitous secondary metabolites in higher plants, characterized by a core 2‐phenylbenzopyrylium cation structure (the anthocyanidin aglycone) (Qi et al. 2023). In vivo, these pigments are predominantly stabilized as anthocyanin glycosides, formed via glycosidic linkage of the aglycone to one or more sugar moieties (Qi et al. 2023). Their structural diversity, stability, and ultimate color are further defined by modifications such as acylation and methylation and are profoundly influenced by the cellular microenvironment, including vacuolar pH and the presence of metal ions or co‐pigments (Kallam et al. 2017). Beyond their role in pigmentation, anthocyanins fulfill vital ecological and physiological functions: they attract pollinators and seed dispersers, act as a screen against photo‐oxidative stress, and bolster plant resilience to abiotic challenges (Grünig et al. 2025). From a human health perspective, dietary anthocyanins are potent antioxidants with demonstrated anti‐inflammatory and cardioprotective properties (Khoo et al. 2017). These health‐promoting properties underscore the nutritional significance of anthocyanin‐rich foods. Notably, fruits from the Rosaceae family, such as strawberries, serve as a major dietary source of these bioactive compounds (Martínez‐Ferri et al. 2023). Therefore, research into anthocyanin metabolism occupies a critical position at the nexus of fundamental plant biology, horticultural science, and nutritional research.

Significant progress has been made in elucidating the core anthocyanin biosynthetic pathway, which originates from phenylalanine and proceeds through a series of enzymatic steps catalyzed by key genes, such as *PAL* (*phenylalanine ammonia‐lyase*), *CHS* (*chalcone synthase*), *DFR* (*dihydroflavonol 4‐reductase*), *ANS* (*anthocyanidin synthase*, *also known as leucoanthocyanidin dioxygenase* [*LDOX*]), *and UFGT* (*UDP‐glucose: flavonoid 3‐O‐glucosyltransferase*) (Sunil and Shetty 2022). However, a comprehensive understanding of its regulation—encompassing biosynthesis, modification, transport, and vacuolar sequestration—within the economically vital and phylogenetically diverse Rosaceae family remains incomplete (Zhang et al. 2025). The biosynthesis and accumulation of anthocyanins in Rosaceae fruits are governed by a sophisticated, multi‐layered regulatory system. This system integrates lineage‐specific transcriptional components—such as variants of the canonical MYB‐bHLH‐WD40 (MBW) complex alongside more recently characterized factors like BBX (B‐box protein) and WRKY (WRKY transcription factor) proteins—with developmental cues, environmental signals (e.g., light, temperature, and sugar levels), and emerging post‐transcriptional and epigenetic mechanisms (Alabd et al. 2022; Hu et al. 2024; Jiang et al. 2019; Zhao et al. 2025). Despite these advances, a comprehensive and integrated understanding of how these diverse regulatory tiers converge to precisely control pigmentation in key Rosaceae crops remains fragmented. To address this gap, this review aims to synthesize current knowledge on the molecular architecture and regulatory networks governing anthocyanin metabolism in these economically vital species. We will critically evaluate the genetic, developmental, and environmental factors that modulate this pathway and discuss how an integrated perspective can inform targeted strategies for the genetic and agronomic enhancement of fruit color and nutritional quality.

## 2 Anthocyanin Biosynthetic Pathway and Post‐Synthetic Fate

### 2.1 Core Biosynthetic Pathway

Anthocyanin biosynthesis constitutes a key branch of plant secondary metabolism. Although the specific types and accumulation patterns of anthocyanins vary among species, the core biosynthetic pathway is largely conserved. Research on this pathway reached a relatively mature stage by the late 20th century and has since been extensively characterized in key Rosaceae fruit crops such as apple, strawberry, and pear (Sun et al. 2023; Zhang et al. 2025).

**FIGURE 1:**
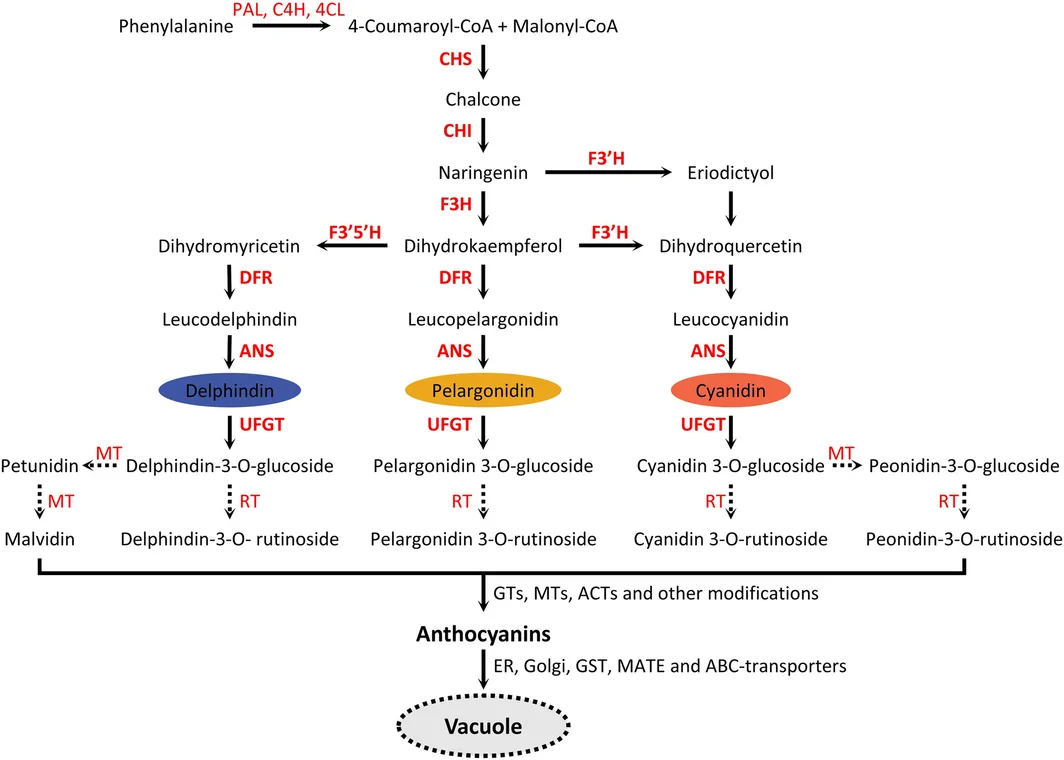
Anthocyanin biosynthetic pathway in Rosaceae fruits. The diagram illustrates the metabolic route from phenylalanine to vacuolar anthocyanins. Key enzymes involved in anthocyanin biosynthesis are highlighted in red. Key steps include: (1) Phenylpropanoid pathway (PAL, C4H, 4CL) generates 4‐coumaroyl‐CoA; (2) Flavonoid backbone synthesis (CHS, CHI, F3H); (3) B‐ring hydroxylation (*F3′H*, *F3′5′H*) directs flux toward three anthocyanidin branches—delphinidin (blue), pelargonidin (orange), and cyanidin (red); (4) Late biosynthesis (DFR, ANS) produces aglycones; (5) Stabilization and diversification via UFGT‐mediated glucosylation and tailoring enzymes (MT, AT, RT); (6) Vacuolar sequestration through ER‐derived vesicles or tonoplast transporters (GSTs, MATE, ABC).

In Rosaceae fruits, the anthocyanin biosynthesis pathway involves a sequential series of reactions catalyzed by key enzymes, which are encoded by a core set of structural genes (Figure 1). The *Phenylalanine ammonia‐lyase* (*PAL*) gene encodes the entry‐point enzyme that catalyzes the deamination of phenylalanine to cinnamic acid, initiating the entire phenylpropanoid pathway. Its expression is closely linked to anthocyanin accumulation in Rosaceae fruits. For instance, members of the *MdPAL* gene family in apple are specifically induced during peel coloration, while the expression of *PcPAL* in pear not only shows a significant positive correlation with anthocyanin content in red‐skinned cultivars but is also strongly modulated by light signaling (Liu, Che, et al. 2013; Yang et al. 2013).

Following this, the *Chalcone synthase* (*CHS*) gene encodes the enzyme responsible for forming the basic carbon skeleton of flavonoids—chalcone. This gene often exists as a gene family in Rosaceae species, exhibiting tissue‐ and developmental stage‐specific expression patterns. The transcripts of *FaCHS* are significantly upregulated during the ripening stage in strawberry (Lunkenbein et al. 2006), and anthocyanin accumulation in the sweet cherry cultivar “Hongdeng” positively correlates with *PacCHS* expression (Liu, Shen, et al. 2013).

The *Chalcone isomerase* (*CHI*) gene encodes the enzyme that catalyzes the cyclization of chalcone to form a key precursor. Although this step is not absolutely essential, *CHI* significantly accelerates the conversion. Its functional relevance in Rosaceae fruits is evident, as *CHI* expression shows a significant positive correlation with anthocyanin accumulation in red pear cultivars such as “Aoguan” and “Mantianhong” (Feng et al. 2008).

Subsequently, the *Flavanone 3‐hydroxylase* (*F3H*) gene encodes a central hub enzyme that catalyzes the formation of dihydroflavonols. As a member of the 2‐oxoglutarate‐dependent dioxygenase family, its role in the coloration of Rosaceae fruits is gaining increased attention. Preliminary studies indicate that the expression dynamics of *F3H* are associated with anthocyanin accumulation patterns during the late developmental stages of fruits like apple and strawberry (Huang et al. 2023; Zhang, Wang, et al. 2023; Zhang, Feng, et al. 2023).

Further branching the anthocyanin pathway, the *Flavonoid‐3′‐hydroxylase* (*F3'H*) gene encodes a cytochrome P450 monooxygenase that introduces a hydroxyl group specifically at the 3′ position of the flavonoid B‐ring (Lin‐Wang et al. 2014; Yanez‐Apam et al. 2023). This modification is pivotal, as it converts dihydrokaempferol into dihydroquercetin, the direct precursor for cyanidin‐type anthocyanins which confer red to purplish‐red hues (Jaakola 2013). In Rosaceae fruits, *F3'H* activity is predominant and closely linked to pigmentation intensity. For instance, comparative studies of apple skin coloration have shown that the expression of *F3'H*, along with other early phenylpropanoid pathway genes, is significantly upregulated in redder bud sport mutants, directly correlating with enhanced anthocyanin accumulation (Li et al. 2018). This establishes *F3'H* as a key enzymatic determinant for the characteristic red spectrum observed across many species within the family.

In contrast, the *Flavonoid‐3′,5′‐hydroxylase* (*F3′5′H*) gene, another cytochrome P450 enzyme, catalyzes the decisive step toward blue‐toned pigmentation by introducing hydroxyl groups at both the 3′ and 5′ positions of the B‐ring (Ma, Tang, et al. 2021). This dual hydroxylation yields dihydromyricetin, the obligatory precursor for delphinidin‐type anthocyanins, which are fundamental to violet and blue colors (Bhosale et al. 2025). Notably, in many economically important Rosaceae fruits such as apple, peach, and raspberry, functional *F3′5′H* activity is typically absent or negligible due to the lack of a functional gene (Zhang et al. 2025). This biochemical constraint explains the general scarcity of delphinidin derivatives and the consequent limitation of the natural color palette in these fruits to shades of red and purple (Sun et al. 2023). Research in raspberry suggests the absence of a functional *F3′5′H* homolog in its genome, underscoring the genetic basis for this color limitation and identifying a clear target for metabolic engineering strategies aimed at expanding color diversity (Sun et al. 2025).

Further downstream, the *Dihydroflavonol 4‐reductase* (*DFR*) gene is crucial, as the substrate specificity of its encoded enzyme is a key determinant of the final anthocyanin type (e.g., pelargonidin, cyanidin, delphinidin) and, consequently, the color phenotype. This selectivity is vital in Rosaceae fruits. The high catalytic efficiency of the apple *MdDFR* gene product toward dihydroquercetin (DHQ) underlies the predominant accumulation of cyanidin‐based glycosides in apple peel. Similarly, the activity of *FaDFR* has been identified as a key limiting factor for red pigmentation in strawberry fruit (Chen et al. 2024; Li, Gao, et al. 2023).

The *Anthocyanidin synthase* (*ANS*) gene, also known as *Leucoanthocyanidin dioxygenase* (*LDOX*), encodes the enzyme that oxidizes colorless precursors into colored anthocyanidins, making it essential for tissue pigmentation. In Rosaceae fruits, its expression displays precise spatial specificity that aligns with pigment distribution, such as its specific high expression in the fruit epidermis of strawberry and raspberry (Feng et al. 2023; Sun et al. 2025). Functional studies confirm its critical role: a frameshift mutation in the *ANS* gene leads to loss of function and a consequent lack of anthocyanin biosynthesis in yellow raspberry (Sargent et al. 2025). In apple, the promoter of the *MdANS* gene contains light‐responsive elements, and its expression is directly regulated by light signals, explaining the deeper coloration on the sun‐exposed side of the fruit (Henry‐Kirk et al. 2018).

Finally, the *UDP‐glucose: flavonoid 3‐O‐glucosyltransferase* (*UFGT*) gene encodes the enzyme that stabilizes anthocyanins through glycosylation, often considered the final “switch” in the accumulation process (Muhammad et al. 2022). In Rosaceae fruits, the activity of this gene frequently constitutes the decisive last step for coloration. The expression of *MdUFGT* is a major molecular marker distinguishing red from green apple cultivars (Ao et al. 2025), and coloration in strawberry strictly depends on the activation of *FaUFGT* during ripening (Yuan et al. 2022). Furthermore, specific *UFGT* gene isoforms contribute to structural diversity by catalyzing unique glycosylation patterns, such as initiating the formation of acylated anthocyanins in certain red‐fleshed peach cultivars (Kou et al. 2023).

In Rosaceae fruit trees, the core anthocyanin biosynthetic genes show a pattern of conserved function coupled with regulatory diversification. Critical enzyme domains (e.g., in CHS, DFR, UFGT) are preserved across species, reflecting strong evolutionary constraint on core biochemistry (Koes et al. 2005). This conservation, however, coincides with significant divergence in gene copy number, expression patterns, and promoter cis‐elements. Instances include divergent *CHS/DFR* expression during fruit development in strawberry versus apple, and differential light response of *PpUFGT* in peach compared to pear (Castillejo et al. 2020; Zhou et al. 2015). Consequently, orthologous genes from different genera cluster separately in phylogenies, suggesting that independent adaptive evolution after species divergence has shaped traits related to fruit development and environmental adaptation (Shulaev et al. 2011; Verde et al. 2013).

### 2.2 Metabolic Flux and Branch Point Control

Metabolic flux through the flavonoid pathway in Rosaceae fruits is governed by a multi‐tiered regulatory architecture encompassing enzyme complex assembly, branch‐point competition, feedback inhibition, and vacuolar sink capacity. At the entry point, PAL and C4H co‐localize on microsomal membranes to form a metabolon that channels carbon into the phenylpropanoid grid, thereby restricting flux into competing lignin or volatile routes (Achnine et al. 2004). Downstream, the partitioning of dihydroflavonols between flavonols and anthocyanins is determined by the competitive activities of FLS and DFR; in *Rubus chingii*, flavonol accumulation exerts product inhibition on DFR, creating a self‐limiting feedback loop that dynamically balances flux between the two branches (Lei et al. 2023). Transcriptional feedback further modulates flux: in pear, ethylene‐activated *PpERF105* induces the repressor PpMYB140, which sequesters PpbHLH3 into non‐productive heterodimers and thereby dismantles the activator MBW complex (Ni et al. 2021), whereas apple MdMYB306‐like directly binds MdMYB17 and MdbHLH33 to sterically obstruct activator complex assembly (Wang, Zhang, et al. 2022). Ultimately, sink capacity frequently constitutes the rate‐limiting step; in grapevine, antisense suppression of either GST or anthoMATE transporters blocks vacuolar accumulation despite intact upstream biosynthesis, demonstrating that the rate of vesicle‐mediated sequestration—not enzymatic capacity—determines net pigment yield (Gomez et al. 2011). Collectively, these findings indicate that Rosaceae fruit coloration is controlled by an integrated flux‐control network in which channeling, feedback, and vacuolar trafficking converge to set the final anthocyanin output.

### 2.3 Post‐Synthetic Modifications

As illustrated in Figure 1, the vivid and stable coloration of Rosaceae fruits is ultimately defined not by anthocyanin biosynthesis alone, but by a mandatory sequence of post‐synthetic modifications—glycosylation, methylation, and acylation (Jaakola 2013). These enzymatic transformations collectively convert chemically unstable anthocyanidin precursors into the diverse and resilient pigments that determine the fruit's final hue, stability, and subcellular fate (Cappellini et al. 2021).

Glycosylation constitutes a foundational and diversified post‐synthetic modification essential for anthocyanin stability in Rosaceae fruits. This process is initiated by the core glucosylation catalyzed by UDP‐glucose: flavonoid 3‐O‐glucosyltransferases (UFGTs), a step often serving as a rate‐limiting bottleneck for pigment accumulation. For instance, functional validation in sweet cherry demonstrated that *Agrobacterium*‐mediated overexpression of *PavUFGT* markedly enhanced fruit pigmentation (Muhammad et al. 2022). This phenotypic change was concomitant with a significant upregulation of two key anthocyanins, cyanidin‐3‐O‐glucoside and cyanidin‐3‐O‐rutinoside. In contrast, virus‐induced gene silencing (VIGS) of *PavUFGT* produced the opposite effect, confirming its essential role in coloration (Pei et al. 2025). Subsequently, secondary glycosylation, such as rhamnosylation catalyzed by rhamnosyltransferases (e.g., forming rutinosides), further enhances pigment stability and structural diversity (Ono et al. 2010). These modification patterns exhibit notable species specificity. In pear, the major anthocyanin is identified as cyanidin‐3‐O‐galactoside (Du et al. 2025); in strawberry, both pelargonidin‐3‐O‐glucoside and its rhamnosylated derivatives are present (Nowicka et al. 2019); and in Chinese plum, peonidin‐3‐O‐rutinoside is detected in the fruit skin (Dai et al. 2024). Collectively, this intricate glycosylation network establishes the molecular basis for the rich and varied coloration of Rosaceae fruits.

Methylation mediated by O‐methyltransferases (OMTs) fine‐tunes the color spectrum by modifying the anthocyanidin B‐ring (particularly at the 3′ position), shifting hues toward redder or bluer tones depending on the degree of substitution (Jaakola 2013). Species‐specific OMT activities, such as the distinct substrate preferences found in peach ( *Prunus persica* ) versus blackberry (*Rubus* spp.), contribute to the distinct color profiles of different fruits (Cheng et al. 2014; Moyer et al. 2002). This modification also serves as a dynamic interface with the environment (Xiangzhao et al. 2026). A prime example is demonstrated in Chinese plum ( *Prunus salicina* ), where integrated metabolomic and transcriptomic analyses reveal that light‐responsive OMT genes coordinate the accumulation of purplish peonidin derivatives (Xiong et al. 2025). Research indicates that post‐harvest light and temperature treatments can significantly induce the expression of master regulators like PsMYB10.1 (or PwMYB10 homologs), which in turn activate the methylation network, promoting the synthesis of these methylated anthocyanins in the fruit skin (Fang et al. 2021). Metabolomic comparisons suggest that differential OMT activity—producing primarily peonidin‐based anthocyanins in peach versus more highly substituted malvidin/petunidin derivatives in blackberries—underlies the classic color contrast between these two Rosaceae fruits (Moyer et al. 2002; Sun et al. 2023).

Acylation catalyzed by enzymes of the BAHD family dramatically enhances the pigment's practical durability (Moghe et al. 2023; Yanez‐Apam et al. 2023). This modification, involving the addition of aromatic acyl groups like caffeic acid, improves resistance to heat, light, and pH changes through intramolecular copigmentation (Kong et al. 2023; Yanez‐Apam et al. 2023). The biotechnological potential of this step is significant. The recent discovery of BAHD acyltransferases capable of producing exceptionally stable caffeoylated anthocyanins in raspberry fruits highlights direct targets for molecular breeding (Teng et al. 2022). This potential is being actively realized in synthetic biology; landmark proof‐of‐concept studies have successfully reconstructed complete anthocyanin pathways in *E. coli* , enabling fermentative production and showcasing sustainable routes to high‐value natural colorants (Jones et al. 2017). In strawberry, a recent 2025 study demonstrated that suppressing the *FaHMGR* gene led not only to increased total anthocyanin content but also to the emergence of five novel anthocyanin components, suggesting that upstream metabolic perturbations can reshape the acylation landscape and create new pigment diversity (Zheng et al. 2025).

In conclusion, the precise chemical assembly line of glycosylation, methylation, and acylation is fundamental to the color quality of Rosaceae fruits. Contemporary research, leveraging gene editing, advanced enzymology, and synthetic biology, is rapidly translating this fundamental knowledge into powerful applications for precision breeding and the bio‐manufacturing of next‐generation, stable pigments for the food and agricultural industries.

### 2.4 Transportation and Storage

Following biosynthesis, anthocyanins are selectively imported into the vacuole through specialized, energy‐dependent mechanisms. In addition to direct cytosolic transport, anthocyanins or their precursors may be trafficked to the vacuole via vesicles derived from the endoplasmic reticulum (ER), the Golgi apparatus, or through autophagy‐related pathways, as depicted in Figure 1 (Zhao 2015). More commonly, two primary and complementary molecular models are recognized. The first involves glutathione S‐transferases (GSTs) acting as cytoplasmic carriers or ligandins that facilitate substrate delivery to the tonoplast (Buhrman et al. 2022). In apple, the functional loss of MdGSTU12 severely impairs anthocyanin accumulation in the peel (Zhao et al. 2021). Similarly, reduced anthocyanins in petioles codes for a GST anthocyanin transporter that is essential for the foliage and fruit coloration in strawberry (Luo et al. 2018). The second model features direct translocation across the tonoplast mediated by specific transporter families. Key players include Multidrug and Toxic Compound Extrusion (MATE) transporters and ATP‐Binding Cassette (ABC) transporters (Buhrman et al. 2022). For instance, the MATE transporter MdMATE1 in apple localizes to the vacuolar membrane and is implicated in flavonoid transport (Frank et al. 2011). Concurrently, transcriptomic analyses in pear associate the upregulation of specific ABC transporter genes with anthocyanin accumulation, suggesting their contributory role in vacuolar sequestration (Kou et al. 2024).

Once sequestered, the final color displayed by anthocyanins is governed by the physicochemical conditions of the intravacuolar milieu (Yanez‐Apam et al. 2023). Vacuolar pH stands as the principal determinant, maintained by proton pumps such as the H^+^‐ATPase (MdVHA) and H^+^‐PPase (MdVP) in apple (Hu et al. 2016). The upregulation of these pumps during fruit reddening correlates with a more acidic lumen and the stabilization of the red flavylium cation form (Hu et al. 2016). Copigmentation further refines the color outcome through non‐covalent interactions. Co‐localized compounds, such as flavonols in strawberry or chlorogenic acid in red‐fleshed apples, interact with anthocyanins to enhance color stability, intensity, and depth, often shifting hues toward deeper purplish‐red tones (Bouillon et al. 2025; Er An et al. 2022).

Therefore, fruit coloration integrates two critical stages: targeted vacuolar import and intravacuolar color stabilization. A molecular understanding of this continuum functionally links biosynthetic pathways to the ultimate phenotypic trait, providing a strategic framework for the targeted improvement of fruit color quality.

## 3 Transcriptional Regulation

Beyond the core structural genes, the biosynthesis of anthocyanins in Rosaceae fruits is governed by a sophisticated, multi‐layered transcriptional regulatory network (Zhang et al. 2025). Various transcription factors (TFs) coordinate to activate or repress the expression of these structural genes, thereby precisely modulating pigment production, as detailed in Table 1.

**TABLE 1:** Transcription factors involved in the regulation of anthocyanin biosynthesis in major Rosaceae fruit crops.

Species	Transcription factors	Function	Target genes	Interacting proteins	Influencing factors	References
*Malus domestica*	MdMYB305	Negatively	*F3H*, *DFR*, *UFGT*	bHLH33		DOI: 10.1111/tpj.16100
*Malus domestica*	MdMYB21	Negatively	*AGT1*, *GSTU1/16/22*			DOI: 10.1016/j.plaphy.2025.110806
*Malus domestica*	MdMYB24L	Positively	*DFR*, *UFGT*	JAZ8/11, MYC2	Methyl jasmonate	DOI: 10.1016/j.plaphy.2019.03.031
*Malus domestica*	MdMYB306‐like	Negatively	*DFR*, *MYB17*	bHLH33, MYB17		DOI: 10.1111/tpj.15720
*Malus domestica*	MdMYB6	Negatively	*ANS*, *GSTF12*	TMT1		DOI: 10.1038/s41438‐020‐0294‐4
*Malus domestica*	MdbHLH3	Positively	*DFR*, *UFGT*	MYB1	Low temperature	DOI: 10.1111/j.1365‐3040.2012.02523.x
*Malus domestica*	MdbHLH33	Positively	*MYBPA1*		Low temperature	DOI: 10.1111/tpj.14013
*Malus domestica*	MdbHLH162	Negatively	*bHLH3/33*	RGL2a, JAZ1/2	Gibberellin, jasmonic acid	DOI: 10.1111/jipb.13608
*Malus domestica*	MdWRKY75	Positively	*MYB1*			DOI: 10.1071/fp21146
*Malus domestica*	MdWRKY71‐L	Positively	*MYB1*, *UFGT*, *HY5*		Ultraviolet‐B	DOI: 10.1016/j.envexpbot.2022.105000
*Malus domestica*	MdWRKY17	Positively	*CHS*	WRKY50	Drought stress	DOI: 10.1016/j.plantsci.2023.111965
*Malus domestica*	MdNAC1	Positively	*MYB10*, *UFGT*	bZIP23	Abscisic acid (ABA)	DOI: 10.1093/hr/uhad049
*Malus domestica*	MdNAC33	Positively	*bHLH3*, *DFR*, *ANS*	MYB1	5‐Aminolevulinic acid (ALA)	DOI: 10.1016/j.plantsci.2023.111949
*Malus domestica*	MdNAC42	Positively		MYB10		DOI: 10.1093/treephys/tpaa004
*Malus domestica*	MdNAC52	Positively	*MYB9/11*	HY5	Light	DOI: 10.1016/j.plantsci.2019.110286
*Malus domestica*	MdT5H5	Positively		bZIP23	Light	DOI: 10.1093/plphys/kiaf477
*Malus domestica*	MdPIF1	Positively	*PAL*, *F3H*		Viroid‐derived small interfering RNA	DOI: 10.1111/pce.15051
*Malus domestica*	MdERF1B	Positively	*MYC2*	JAZ5/10	Ethylene, jasmonic acid	DOI: 10.1093/hr/uhac142
*Malus domestica*	MdARF19	Negatively	*LOB52*		Nitrogen	DOI: 10.1007/s00344‐017‐9766‐7
*Malus domestica*	MdGATA15	Negatively	*MYB11/308*, *ANS*, *GSTF12*	PIF4‐Like3	High temperature	DOI: 10.1111/tpj.70381
*Malus domestica*	MdROS1	Positively	*F3'H*, *UFGT*		Low temperature	DOI: 10.1093/hr/uhac007
*Pyrus* spp.	PuMYB93	Positively	*GSTF12*			DOI: 10.1016/j.jia.2025.12.002
*Pyrus* spp.	PpERF9	Negatively		MYB114, RAP2.4		DOI: 10.1093/plcell/koad077
*Pyrus* spp.	PpERF105	Negatively	*MYB140*		Ethylene	DOI: 10.1111/tpj.15049
*Pyrus* spp.	PpbHLH64	Positively	*MYB10*		Light	DOI: 10.1104/pp.20.01188
*Pyrus* spp.	Pp4ERF24, Pp12ERF96	Positively		MYB114	Blue light	DOI: 10.1007/s11103‐018‐0802‐1
*Pyrus* spp.	PyWRKY40	Negatively	*DFR*		Salicylic acid (SA)	DOI: 10.1016/j.plantsci.2024.112323
*Pyrus* spp.	PyWRKY26	Positively	*MYB114*	bHLH3		DOI: 10.1038/s41438‐020‐0254‐z
*Fragaria* spp.	FvCSN5	Dual‐function	*CHS*, *F3H*	MYB1, BBX20		DOI: 10.1111/tpj.70021
*Fragaria* spp.	FaMYB10L	Positively	*RAP*	bHLH3	Light	DOI: 10.3390/ijms242316561
*Fragaria* spp.	FaMYB5	Positively	*PAL*, *C4H*, *4CL*	EGL3, LWD1/1‐like	lncRNA‐myb5	DOI: 10.1111/pbi.14024
*Fragaria* spp.	FaMYB5	Positively	*F3'H、4CL‐2、TT12*	BBX24		DOI: 10.3390/ijms241512185
*Fragaria* spp.	FvbHLH9	Positively	*DFR*	HY5	Light	DOI: 10.1093/pcp/pcaa010
*Fragaria* spp.	FvMAPK3	Negatively	*MYB10*, *CHS*		Low temperature	DOI: 10.1093/plcell/koac006
*Fragaria* spp.	FvCOP1	Negatively	*HY5*, *RI*, *MYB10*		Light	DOI: 10.1093/plphys/kiaf339
*Prunus persica*	PpNAP4	Positively	*ANS*, *MYB10.1*			DOI: 10.1021/acs.jafc.4c03924
*Prunus persica*	PpMYB39	Positively	*DFR*			DOI: 10.3390/horticulturae8040332
*Prunus persica*	PpHY5	Positively	*MYB10.1*	BBX10		DOI: 10.1111/tpj.16189
*Prunus avium*	PavMYB10.1	Positively	*ANS*, *UFGT*	PavbHLH, PavWD40		DOI: 10.1111/pbi.12568
*Prunus avium*	PavMYB.C2	Positively	*UFGT*			DOI: 10.1371/journal.pgen.1011761
*Prunus avium*	PavbHLH102	Positively	*F3H*, *F3'H*, *DFR*, *UFGT*			DOI: 10.1016/j.hpj.2025.07.007
*Prunus avium*	PavBBX6/9	Positively	*UFGT*		Light, ABA	DOI: 10.1093/plphys/kiad137
*Rubus* spp.	RuHCT1	Positively				DOI: 10.1016/j.scienta.2024.113541
*Rubus* spp.	RuMYB10	Positively				DOI: 10.1016/j.scienta.2012.04.025

*Note:* The table summarizes key transcription factors (TFs) that regulate structural genes in the anthocyanin pathway across economically important Rosaceae fruits. Species: The common and scientific names of the fruit crop. Transcription factors: The abbreviated name of the TF. Function: “Positively” or “Negatively” indicates whether the TF activates or represses the anthocyanin biosynthesis. Target genes: Structural or regulatory genes directly modulated by the TF. Interacting proteins: Other proteins (e.g., from MBW complex) that interact with the TF to exert its function. Influencing factors: Developmental, hormonal, or environmental cues known to modulate the TF's activity or expression. References: Key supporting literature, provided as Digital Object Identifiers (DOIs).

### 3.1 The Core MBW Regulatory Module

**FIGURE 2:**
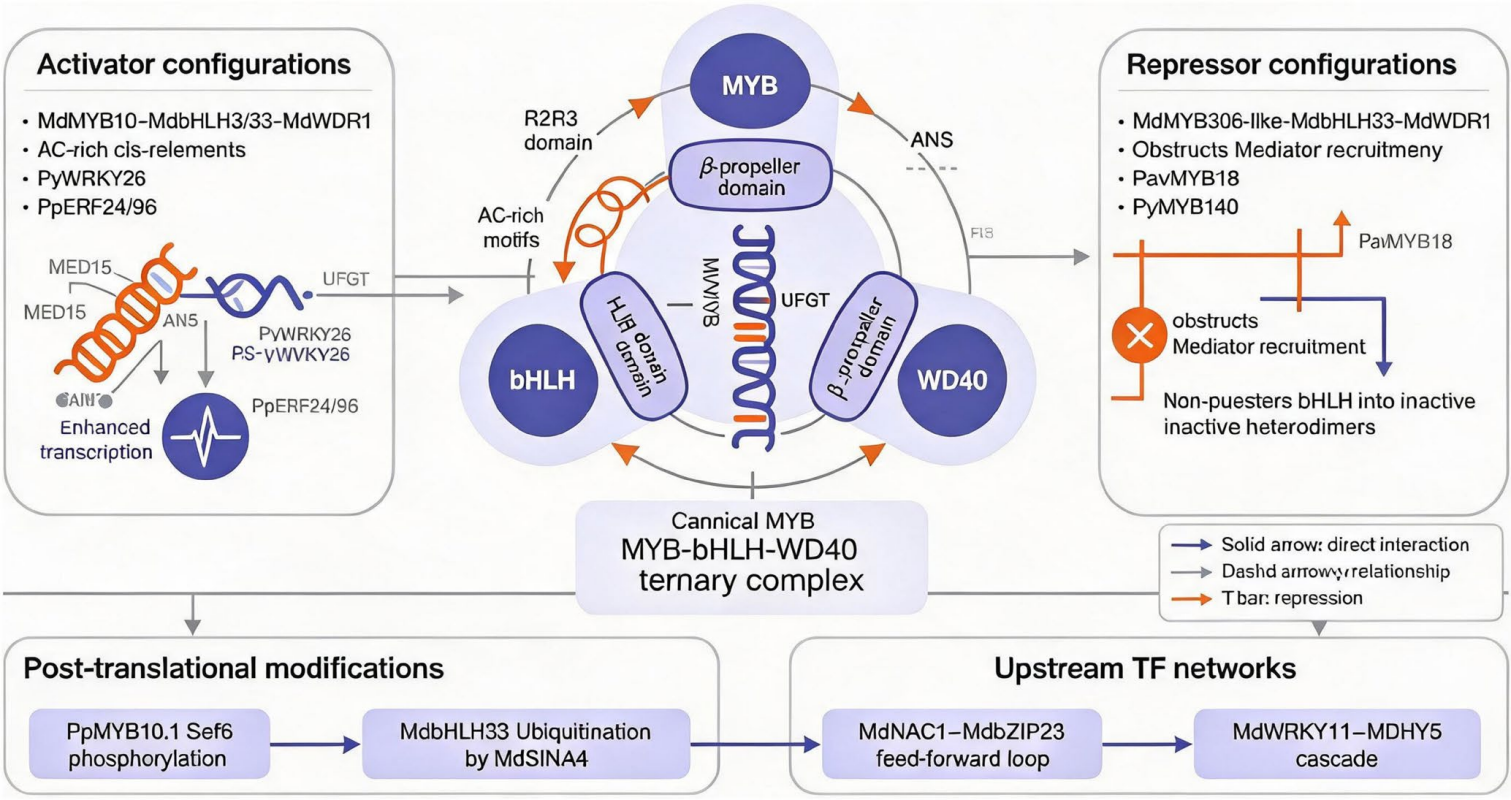
Conceptual model of MBW transcriptional regulation. Schematic of the MYB–bHLH–WD40 ternary complex and its regulatory configurations. Central panel: domain architecture (MYB R2R3 DNA‐binding; bHLH HLH bridging; WD40 β‐propeller stabilization) and target promoter recognition. Left: activator complexes (MdMYB10–MdbHLH3/33–MdWDR1) with co‐activators (MED15) and accessory factors (PyWRKY26, PpERF24/96). Right: repressor complexes (MdMYB306‐like, PavMYB18, PyMYB140) operating through Mediator obstruction, non‐productive DNA binding, and bHLH sequestration, respectively. Bottom: post‐translational switches (PpMYB10.1 phosphorylation, MdbHLH33 ubiquitination) and upstream TF integration (MdNAC1–MdbZIP23, MdWRKY11–MdHY5). Solid arrows, direct interactions; dashed arrows, regulatory relationships; T‐bars, repression.

The MYB–bHLH–WD40 (MBW) complex serves as the central transcriptional hub for anthocyanin biosynthesis in Rosaceae (Ramsay and Glover 2005). Figure 2 illustrates its hierarchical architecture: the MYB R2R3 domain binds AC‐rich cis‐elements in target promoters (e.g., *ANS*, *UFGT*); the bHLH HLH domain bridges MYB–WD40 interactions; and WD40 β‐propeller domains stabilize the complex through hydrophobic contacts (Ramsay and Glover 2005). This core module is exemplified by the MdMYB10–MdbHLH3/33–MdWDR1 complex in apple (Espley et al. 2007). Accessory factors further potentiate activator output by reinforcing complex recruitment to chromatin. In pear, PyWRKY26 cooperates with PybHLH3 to co‐target the *PyMYB114* promoter (Li et al. 2020), whereas PpERF3 interacts directly with PpMYB114 and PpbHLH3 to assemble a functional ERF3–MYB114–bHLH3 complex (Yao et al. 2017). Likewise, Pp4ERF24 and Pp12ERF96 strengthen MBW complex formation to promote blue light‐induced anthocyanin accumulation in red‐skinned pear fruits (Ni et al. 2019).

Repressor configurations antagonize activator output through distinct molecular strategies. Dominant‐negative inhibition occurs when MdMYB306‐like binds MdMYB17 and MdbHLH33, sterically obstructing activator complex assembly (Wang, Luo, et al. 2022). Competitive sequestration of shared bHLH partners provides an additional layer of attenuation: ethylene‐activated PpERF105 transcriptionally induces PpMYB140, which subsequently competes with PpMYB114 for PpbHLH3 binding, forming inactive heterodimers that dismantle the activator complex (Ni et al. 2021). Similarly, apple MdMYB16 interacts with MdbHLH33 to attenuate anthocyanin accumulation (Xu et al. 2017), whereas in peach, PpMYB18 functions as a repressor‐type R2R3‐MYB that is induced by activator‐type MYBs to balance anthocyanin and proanthocyanidin accumulation, thereby establishing a negative feedback loop (Zhou et al. 2019).

Beyond complex stoichiometry, post‐translational modifications and upstream transcriptional networks dynamically modulate MBW stability and transcriptional output. In apple, the E3 ubiquitin ligases MdSINA4 and MdSINA11 antagonistically regulate anthocyanin biosynthesis by targeting the IAA29–ARF5‐1–ERF3 module for ubiquitination‐dependent degradation, thereby modulating auxin–ethylene crosstalk and downstream MBW activity (Li, Liu, et al. 2023). Environmental and developmental cues are integrated through hierarchical TF cascades: the ABA‐inducible NAC transcription factor MdNAC1 interacts with MdbZIP23 to activate *MdMYB10* and *MdUFGT* transcription in red‐fleshed apples (Liu, Mei, et al. 2023), whereas MdWRKY11 binds W‐box cis‐elements in the promoters of *MdMYB10*, *MdMYB11*, and *MdHY5* to enhance the activity of the photoresponse factor MdHY5 and promote pigment accumulation (Liu, Wang, et al. 2019).

### 3.2 Extended Transcription Factor Families in Direct Regulation of Structural Genes

Beyond the canonical MBW hub, several transcription factor families outside the MYB–bHLH–WD40 triad directly bind to the promoters of anthocyanin biosynthetic genes, providing independent or synergistic inputs that fine‐tune pathway output in response to environmental and developmental cues.

The WRKY family directly targets multiple structural loci. In apple, MdWRKY11 binds W‐box cis‐elements in the *MdUFGT* promoter to promote anthocyanin glycosylation and also activates *MdMYB10* and *MdMYB11* to amplify the transcriptional cascade (Liu, Su, et al. 2019). MdWRKY40 directly binds the W‐box in the *MdANS* promoter and physically interacts with MdMYB1 to enhance the latter's affinity for downstream targets such as *MdDFR* and *MdUF3GT* (An et al. 2019). In pear, PbWRKY75 promotes anthocyanin accumulation by directly binding to and activating the *PbDFR* and *PbUFGT* promoters, recruiting the activator PbMYB10b as a co‐regulator (Cong et al. 2021).

AP2/ERF and bZIP proteins provide additional direct inputs. The strawberry RAV transcription factor FaRAV1 directly binds to and transactivates the promoters of *CHS*, *F3H*, *DFR*, and *GT1*, while also up‐regulating *FaMYB10* to reinforce the MBW‐driven positive loop (Zhang et al. 2020). In pear, PybZIPa stimulates *PyUFGT* expression by interacting with tandem G‐box cis‐elements in its promoter, with transactivation potency dependent on G‐box copy number (Liu, Su, et al. 2019).

### 3.3 Indirect Regulation and Combinatorial Interaction Networks

Transcription factors also orchestrate anthocyanin synthesis indirectly by targeting upstream regulatory genes or components outside the core pathway, thereby creating hierarchical and feedback‐driven networks. For instance, apple ALA‐induced MdBBX47 directly binds G‐box motifs in the *MdCHS* and *MdUFGT* promoters while also transcriptionally activating *MdMYB110a*, which subsequently upregulates the transport gene *MdGSTF12*; this dual direct‐and‐indirect architecture positions MdBBX47 as an upstream node that integrates into both MBW and non‐MBW transcriptional cascades (Iqbal et al. 2026). Similarly, MdMYB110a interacts with MdWD40‐280 and MdHsfB3a to enhance its own transactivation capacity on *MdGSTF12*, and MdHsfB3a can independently bind the *MdDFR* and *MdANS* promoters, revealing a cooperative network in which accessory factors both potentiate and bypass the core MBW module (Fang et al. 2023). In strawberry, the B‐box protein FaBBX22 cooperates with FaHY5 to enhance the latter's DNA‐binding affinity to G‐box elements in the promoters of anthocyanin structural genes, thereby indirectly promoting transcriptional activation beyond the capacity of FaHY5 alone (Liu et al. 2022). Similarly, in Japanese plum ( *Prunus salicina* ), the ethylene‐responsive factor PsERF1B integrates cold‐storage signals by interacting with PsMYB10.1 and PsbHLH3 to form a functional PsERF1B–PsMYB10.1–PsbHLH3 module (Xu et al. 2023).

Hierarchical amplification loops also operate through metabolic–transcriptional feedback. In sweet cherry, light‐induced PavBBX6 and PavBBX9 directly bind G‐box elements in the *PavUFGT* promoter to promote anthocyanin glycosylation, while simultaneously activating the ABA biosynthetic gene *PavNCED1*. The resulting ABA accumulation further upregulates PavBBX6/9 expression, establishing a positive feedback loop that indirectly sustains and amplifies anthocyanin production (Wang et al. 2023).

Collectively, these multi‐layered mechanisms—encompassing chromatin‐level repression, TF‐mediated hierarchical amplification, and heterodimeric cooperative binding—allow precise integration of hormonal and environmental signals, ultimately determining pigmentation patterns.

## 4 Epigenetic and Post‐Transcriptional Regulation

Beyond transcriptional control by the MBW complex, anthocyanin biosynthesis is subject to additional layers of epigenetic and post‐transcriptional modulation that fine‐tune gene expression with precision and reversibility.

### 4.1 Epigenetic Regulation

DNA methylation and histone modifications constitute critical epigenetic layers that govern anthocyanin pathway genes in Rosaceae without altering the underlying DNA sequence. In apple, differential cytosine methylation across the *MdMYB10* promoter correlates with sectorial pigmentation patterns: red stripes exhibit hypomethylation relative to green stripes, permitting TF access and transcriptional activation, whereas hypermethylated green stripes silence *MdMYB10* expression (Telias et al. 2011). A similar mechanism operates in pear, where increased methylation at the *PcMYB10* promoter attenuates pigment synthesis, and light‐induced debagging triggers demethylation of the *PcUFGT* promoter via upregulation of DNA demethylases, thereby licensing rapid coloration upon illumination (Liu, Shu, et al. 2023). In peach, low‐temperature‐induced anthocyanin accumulation is preceded by active demethylation at multiple biosynthetic loci—including *PpPAL1/2*, *PpC4H*, *PpF3H*, *PpDFR*, and *PpANS*—mediated by DNA demethylases and methyltransferases, establishing a temperature–epigenetic axis that links environmental sensing to pigment production (Zhu et al. 2020).

Histone modifications operate in parallel to modulate chromatin accessibility. In crabapple (*Malus crabapple*), virus‐induced silencing of the histone deacetylase *McHDA6* reduces transcription of the DNA methyltransferase *McMET1* and derepresses *McMYB10*, leading to elevated anthocyanin content; this illustrates crosstalk between histone deacetylation and DNA methylation machineries in maintaining the repressive chromatin state of anthocyanin regulators (Peng et al. 2020). At the nucleosome level, the histone H2A variant H2A.Z antagonizes the activating mark H3K4me3 at anthocyanin biosynthetic genes: H2A.Z deposition prevents H3K4me3 accumulation and maintains a repressive chromatin environment, whereas H2A.Z deficiency permits H3K4me3 enrichment and licenses gene expression (Cai et al. 2019). In strawberry fruit, ripening‐related genes acquire activating H3K4me3 and H3K9ac marks while losing repressive H3K27me3, indicating that dynamic histone modification remodeling serves as a developmental switch for pigment production (Pan et al. 2024). Collectively, these multi‐layered epigenetic mechanisms—DNA methylation, histone acetylation, and histone methylation—integrate developmental cues and environmental signals to fine‐tune anthocyanin output.

### 4.2 Post‐Transcriptional Regulation

Non‐coding RNAs have emerged as versatile post‐transcriptional regulators of anthocyanin biosynthesis in Rosaceae, operating through microRNA (miRNA)‐mediated cleavage, endogenous target mimicry, and long non‐coding RNA (lncRNA)‐directed transcriptional scaffolding. In apple, the lncRNAs MLNC3.2 and MLNC4.6 function as endogenous target mimics (eTMs) of miR156a; by sequestering miR156a, they prevent its cleavage of *SPL2‐like* and *SPL33* transcripts, thereby derepressing SPL‐mediated activation of anthocyanin accumulation during light‐induced fruit coloration (Yang et al. 2019). Another apple lncRNA, MdLNC499, is transcriptionally activated by MdWRKY1 and subsequently induces the expression of *MdERF109*, which directly binds anthocyanin‐related gene promoters to promote early‐stage pigment synthesis (Ma, Yang, et al. 2021).

In strawberry, post‐transcriptional control operates through antisense lncRNA‐mediated repression of transcriptional activators. The R2R3‐MYB factor FaMYB5 positively regulates anthocyanin and proanthocyanidin biosynthesis by activating *F3′H* and *LAR* expression; however, its transcript is negatively regulated by the antisense lncRNA‐myb5, establishing a lncRNA–TF axis that fine‐tunes flavonoid output without altering the core MBW module (Jiang et al. 2023). Additionally, the lncRNA FRILAIR acts as a non‐canonical target mimic of miR397 during fruit ripening, competitively reducing miR397‐mediated repression of *LAC11a* and thereby promoting the expression of anthocyanin biosynthetic genes (Tang et al. 2021).

In sweet cherry, integrated transcriptome and lncRNA analyses have identified extensive lncRNA–mRNA regulatory modules that drive ripening‐associated anthocyanin accumulation. Differentially expressed lncRNAs target key anthocyanin biosynthetic genes—such as *CHS1‐like*, *CHI*, and *DFR*—as well as transcription factor genes including *MYB10.1*, *bHLH33*, and *WD40*, forming a multidimensional post‐transcriptional network that coordinates spatio‐temporal pigment production during the transition from yellow to dark red stages (Liu et al. 2024).

Collectively, these findings illustrate that lncRNA‐mediated post‐transcriptional mechanisms—encompassing miRNA sequestration, antisense repression, and target mimicry—form an integrated regulatory layer that operates downstream of, and in feedback with, transcriptional control, enabling precise spatiotemporal patterning of anthocyanin accumulation in Rosaceae fruits.

## 5 Environmental Regulation

**FIGURE 3:**
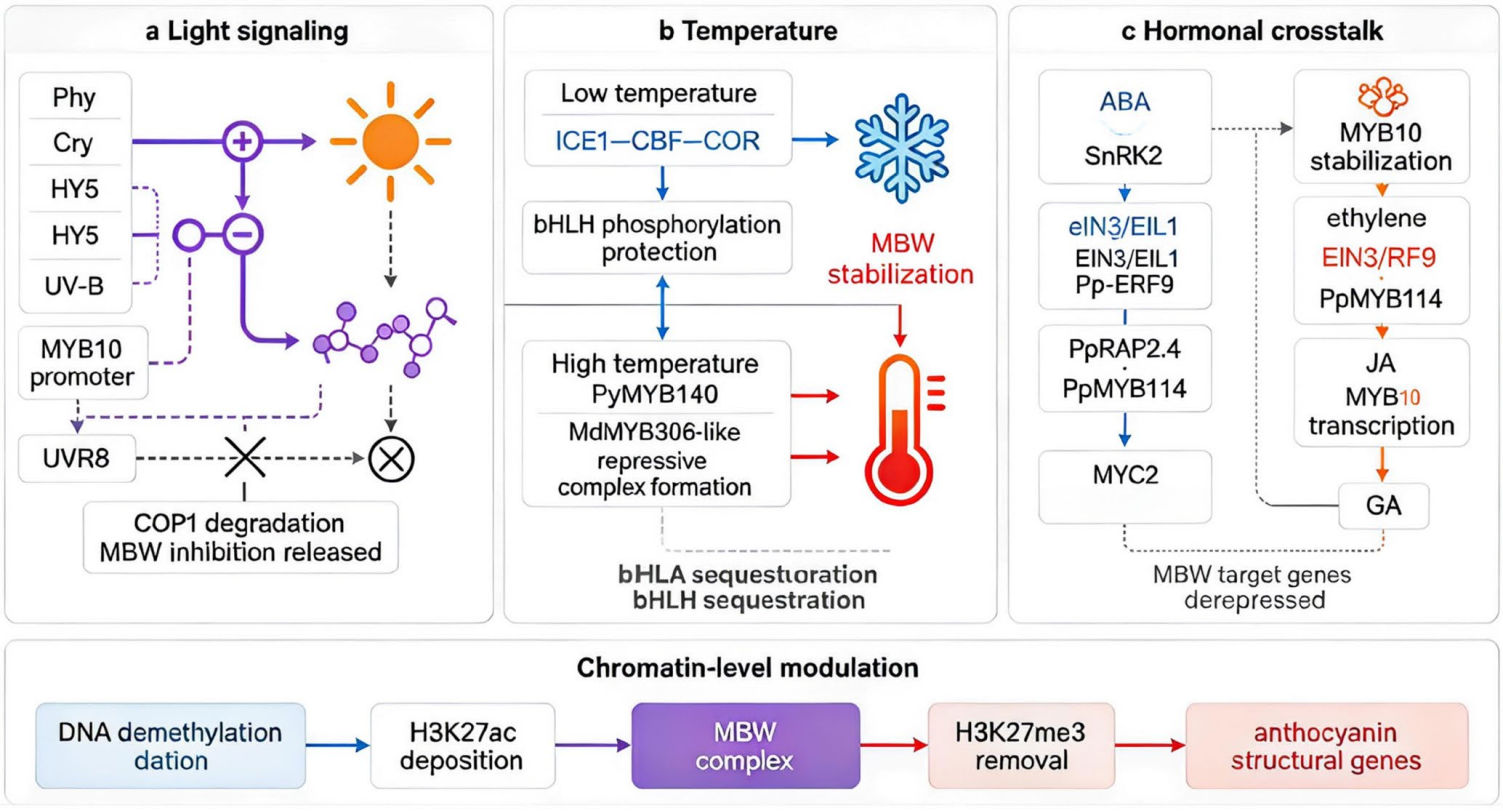
Environmental regulation of anthocyanin biosynthesis. Three signaling modules transduce environmental inputs to the MBW complex. (a) Light: Phy/Cry–HY5 activates MYB10 transcription; UVR8–COP1 degradation releases MBW inhibition. (b) Temperature: low temperature stabilizes MBW via ICE1–CBF–COR–mediated bHLH phosphorylation; high temperature induces PyMYB140 and MdMYB306‐like to form repressive complexes and sequester bHLH partners. (c) Hormones: ABA–SnRK2 phosphorylates and stabilizes MYB10; ethylene–EIN3/EIL1–PpERF9 suppresses PpRAP2.4–PpMYB114; JA–MYC2 activates *MYB10*; GA–DELLA degradation derepresses targets. All modules converge on chromatin remodeling (DNA demethylation, H3K27ac deposition, H3K27me3 removal).

As illustrated in Figure 3, anthocyanin pigmentation in Rosaceae fruits is dynamically modulated by environmental inputs, among which light, temperature, and phytohormones represent the principal exogenous cues. These signals are perceived by specialized sensory and signaling systems and transduced into integrated transcriptional and epigenetic networks that ultimately converge on the core MBW regulatory complex, thereby orchestrating the precise spatiotemporal control of pigment biosynthesis.

### 5.1 Light

Light serves as the most critical inductive signal for anthocyanin accumulation across Rosaceae. In pear, the B‐box protein PpBBX16 is rapidly induced by white‐light irradiation and interacts with PpHY5 to trans‐activate the *PpMYB10* promoter, thereby integrating the COP1–HY5–MYB10 signaling axis into light‐dependent coloration (Bai et al. 2019). In sweet cherry, PavBBX6 and PavBBX9 function as dual‐output sensors: they directly bind G‐box elements in the *PavUFGT* promoter to promote anthocyanin glycosylation, while simultaneously activating the ABA biosynthetic gene *PavNCED1*, establishing a positive feedback loop that sustains light‐induced pigmentation (Wang et al. 2023). In strawberry, the blue‐light photoreceptor FaCRY1 perceives light and physically interacts with the E3 ubiquitin ligase FaCOP1, inhibiting FaCOP1‐mediated degradation of FaHY5; the stabilized FaHY5 subsequently activates *FaMYB10* and downstream structural genes, bridging photoperception to the MBW pathway (Liu, Tang, et al. 2023) (Figure 3a).

Post‐translational phosphorylation provides an additional layer of light signal amplification. In apple, light‐activated MdMPK6 directly interacts with and phosphorylates MdHY5 at Ser‐36, which prevents MdCOP1‐mediated ubiquitination and proteasomal degradation, thereby enhancing MdHY5 stability and its transcriptional activation of anthocyanin‐related genes (Xing et al. 2023). Light also triggers epigenetic reprogramming: in pear, debagging‐induced illumination promotes active DNA demethylation at the *PcUFGT* promoter via upregulation of DNA demethylases, licensing rapid transcriptional activation upon light exposure (Liu, Mei, et al. 2023).

### 5.2 Temperature

Temperature constitutes a decisive environmental factor that operates antagonistically to light: low temperatures promote coloration, whereas high temperatures inhibit anthocyanin synthesis. In apple, the bHLH transcription factor MdbHLH3 is induced by low temperature (14°C) and directly binds to the promoters of *MdDFR* and *MdUFGT* to activate their expression, thereby promoting anthocyanin accumulation in the peel (Xie et al. 2012). The B‐box protein MdBBX20 functions as an integrator of UV‐B and low‐temperature signals: MdbHLH3 binds low‐temperature‐responsive cis‐elements in the *MdBBX20* promoter, and the induced MdBBX20 subsequently interacts with MdHY5 to enhance *MdMYB1* expression and directly activates *MdANS* and *MdDFR* (Fang, Dong, Yue, Hu, et al. 2019) (Figure 3b).

Conversely, high temperatures suppress anthocyanin biosynthesis through distinct mechanisms. In apple, heat shock transcription factors MdHSF3b and MdHSF4a activate the expression of *MdCOL4* (a B‐box repressor), which forms an inactive MdCOL4–MdHY5 heterodimer that obstructs the transcription of *MdANS*, *MdUFGT*, and *MdMYB1* (Fang, Dong, Yue, Chen, et al. 2019). In peach, low‐temperature‐induced anthocyanin accumulation is preceded by active DNA demethylation at multiple biosynthetic loci—including *PpPAL1/2*, *PpC4H*, *PpF3H*, *PpDFR*, and *PpANS*—mediated by DNA demethylases and methyltransferases, establishing a temperature–epigenetic axis that links environmental sensing to pigment production (Zhu et al. 2020). In Japanese plum ( *Prunus salicina* ), the R2R3‐MYB factor PsMYB10.1 enhances anthocyanin accumulation by upregulating *PsANS*, *PsUFGT*, and *PsGST* at 20°C, whereas 30°C suppresses its activity and induces the repressor PsMYB18, which attenuates PsMYB10.2‐driven pigment synthesis (Fang et al. 2021, 2022).

### 5.3 Hormones

Endogenous hormones intricately synchronize with environmental signals to modulate anthocyanin output. In apple, methyl jasmonate (MeJA) and light signals are integrated through the MdWER–MdERF109–MdJAZ2 module: MdERF109 (a light‐responsive TF) interacts with MdWER (a MeJA‐induced AP2/ERF factor) to form a protein complex that enhances the transcription of *MdCHS*, *MdUFGT*, and *MdbHLH3*, while MdWER simultaneously interacts with the JAZ repressor MdJAZ2 to relieve its inhibition of the pathway, thereby coordinating JA and light inputs (Zhang et al. 2024). Drought stress also induces anthocyanin biosynthesis via the ERF transcription factor MdERF38, which directly activates the *MdMYB1* promoter to promote pigment accumulation under water deficit (An et al. 2020).

Abscisic acid (ABA) functions as a central mediator that bridges hormone and environmental signaling. In strawberry, red‐light irradiation specifically elevates pelargonidin‐based anthocyanins by upregulating ABA biosynthesis genes and downregulating ABA catabolism genes, resulting in increased ABA accumulation that activates FaMYB10 and directs metabolic flux toward the pelargonidin branch (Wang, Luo, et al. 2022). In peach, the ethylene response factor PpERF3 directly binds to the promoters of *PpNCED2* and *PpNCED3* (ABA biosynthetic genes) to stimulate ABA production during ripening, which in turn promotes anthocyanin accumulation (Wang et al. 2019) (Figure 3c). In apple, the ABA‐responsive transcription factor MdABI5 directly binds the *MdbHLH3* promoter and enhances its transcription; the MdABI5–MdMYB1–MdbHLH3 protein complex subsequently activates *MdDFR* expression, demonstrating that ABA signaling directly feeds into the MBW complex to regulate pigment synthesis (An et al. 2021). Exogenous ABA application also stimulates ethylene production in strawberry, which accelerates anthocyanin accumulation, revealing a conserved hormone crosstalk axis across climacteric and non‐climacteric Rosaceae fruits (Li et al. 2011).

Collectively, environmental regulation converts physical and chemical signals into precise transcriptional and epigenetic responses by modulating photoreceptor–HY5 modules, temperature‐responsive bHLH/BBX networks, and hormone‐mediated TF cascades, all of which converge on the MBW complex to determine the timing, intensity, and spatial pattern of fruit pigmentation (Figure 3).

## 6 Species‐Specific Regulation in Major Rosaceae Fruits

Anthocyanin regulatory architectures exhibit conserved features and genus‐specific innovations across Rosaceae. Genetic background—encompassing MYB10 copy number, promoter architecture, and epigenetic landscape—constitutes a critical internal determinant that shapes accumulation capacity and modulates environmental responsiveness. This section provides a systematic comparative analysis, integrating preceding molecular mechanisms with species‐specific evolutionary contexts. Identical environmental or hormonal treatments elicit divergent phenotypic responses depending on underlying genetic architecture, underscoring the necessity of genotype–environment interactions in mechanistic models of Rosaceae fruit coloration.

### 6.1 The *Malus* (Apple) Model: Duplicated MYB10 Paralogs and Light‐Dependent Epigenetic Switches

Apple represents the most extensively characterized system, distinguished by tandem duplication and promoter rearrangement at the *MYB10* locus. The *MYB10* gene harbors two principal allelic variants: the R1 allele (predominant in white‐fleshed cultivars) contains a single MYB‐binding motif in its proximal promoter, whereas the R6 allele (characteristic of red‐fleshed accessions) carries six direct tandem repeats of a 23‐bp sequence that generates a positive autoregulatory loop. This minisatellite‐like rearrangement enables the MYB10 protein to bind its own promoter and amplify transcription, thereby driving ectopic anthocyanin accumulation in fruit flesh (Espley et al. 2009). A second duplication event, producing *MYB110a* on linkage group 17, arose from the Maloideae whole‐genome duplication and exhibits subfunctionalized expression restricted to the fruit cortex during late maturation, accounting for the “type 2” red‐flesh phenotype distinct from the *MYB10*‐dependent type 1 (Chagné et al. 2013).

Epigenetic regulation further diversifies apple coloration. The *MYB10* promoter undergoes differential DNA methylation across the fruit peel, with red stripes exhibiting hypomethylation relative to green stripes, thereby permitting sectorial transcriptional activation and the classic striped patterns of sun‐exposed surfaces (Telias et al. 2011). Whole‐genome bisulfite sequencing has further revealed that hypomethylation at CG and CHG sites within the *MdMYB10* promoter—and hypermethylation at the *MdbHLH74* promoter—collectively shift the balance toward anthocyanin accumulation during fruit ripening (Li et al. 2019). At the mechanistic level, ARGONAUTE 4 (AGO4)‐mediated RNA‐directed DNA methylation (RdDM) actively maintains *MdMYB10* promoter methylation, establishing a repressive chromatin state that attenuates anthocyanin accumulation; loss of *MdAGO4* function triggers *MdMYB10* upregulation and ectopic pigmentation (Jiang et al. 2020).

### 6.2 The *Pyrus* (Pear) Paradigm: Temperature‐Sensitive Repressor Hierarchies

Pear anthocyanin regulation is characterized by pronounced temperature sensitivity, with high temperatures severely attenuating pigmentation. This phenotype arises from a distinct regulatory architecture in which proteasomal degradation dynamics modulate transcriptional output under thermal stress. Under ambient temperatures, the RING‐type E3 ubiquitin ligase PpATL52 targets the heat shock transcription factor PpHsfB2a for 26S proteasome‐mediated degradation, thereby relieving its repressive effect on the bZIP factor PpHY5L and permitting anthocyanin gene expression. At elevated temperatures, reduced *PpATL52* expression and diminished PpHsfB2a degradation suppress *PpHY5L* expression, leading to rapid transcriptional shutdown of the biosynthetic pathway (Wang et al. 2025).

This thermal attenuation is reinforced by hierarchical repressor modules. Ethylene‐activated PpERF105 transcriptionally induces PpMYB140, which competitively sequesters PpbHLH3 into inactive heterodimers that displace activator complexes from target promoters (Ni et al. 2021). In parallel, PpERF9 recruits histone deacetylase complexes to the promoters of *PpMYB114* and *PpRAP2.4*, establishing a repressive chromatin state that further dampens transcriptional output under stress conditions (Ni et al. 2023). Conversely, light exposure antagonizes temperature suppression through the B‐box protein PpBBX16, which interacts with PpHY5 to trans‐activate the *PpMYB10* promoter, thereby establishing a temperature–light antagonistic axis that determines final peel coloration (Bai et al. 2019).

### 6.3 The *Fragaria* (Strawberry) System: Non‐Climacteric Ripening and Hormone‐Independent MBW Activation

Strawberry exhibits a distinct regulatory logic reflecting its non‐climacteric ripening physiology. Unlike apple and pear, where ethylene drives respiratory climacteric and coloration, strawberry receptacle ripening is initiated by the decline of achene‐derived auxin and subsequently reinforced by ABA and light signaling (Jia et al. 2011; Perotti et al. 2023). The core MBW module, with FaMYB10 as the central R2R3‐MYB activator, is transcriptionally upregulated during maturation and directly activates the promoters of structural genes including *CHS*, *F3H*, *DFR*, and *UFGT*; both silencing and overexpression of *FvMYB10* in diploid and octoploid backgrounds confirm its obligate role in fruit‐specific anthocyanin accumulation (Lin‐Wang et al. 2014).

Natural variation in strawberry color is predominantly governed by allelic diversity at the *MYB10* locus. In the diploid woodland strawberry ( *F. vesca* ) and the cultivated octoploid (*F. × ananassa*), functionally conserved *MYB10* alleles drive pigment synthesis, whereas white‐fruited accessions harbor disruptive mutations—such as a CACTA‐like transposon insertion in the promoter or a frameshift‐inducing insertion in the coding sequence—that abolish protein function (Castillejo et al. 2020; Wang et al. 2020). The octoploid genome carries additional *MYB10* homoeologs (e.g., *FaMYB10‐2*), whose expression can be enhanced by cis‐regulatory insertions, thereby contributing to quantitative variation in pigment intensity across cultivars (Castillejo et al. 2020). Notably, the R1 and R6 autoregulatory promoter motifs that amplify *MYB10* expression in apple are absent in strawberry, indicating that this positive‐feedback mechanism evolved independently in the *Malus* lineage.

Environmental inputs are transduced into the MBW module through post‐translational modifications. Low temperatures delay red coloration by activating the FvSnRK2.6–FvMAPK3 signaling cascade, which phosphorylates FvMYB10 to reduce its transcriptional activity and simultaneously enhances FvKFB1‐mediated ubiquitination and proteasomal degradation of FvCHS1, thereby dampening metabolic flux through the pathway (Mao et al. 2022). This temperature‐sensitive phosphorylation switch illustrates how strawberry integrates environmental stress signals directly into the core anthocyanin regulatory machinery.

### 6.4 The *Prunus* (Peach, Cherry, Plum) Clade: Single‐Locus Control and Dormancy‐Coupled Pigmentation

Stone fruits within *Prunus* exhibit comparatively simpler regulatory architectures, typically governed by single *MYB10* loci without the extensive tandem duplication seen in apple. In peach ( *P. persica* ), multiple *PpMYB10* paralogs (*PpMYB10.1–10.4*) display tissue‐specific expression patterns: *PpMYB10.1–10.3* regulate fruit pigmentation, whereas *PpMYB10.4* controls leaf coloration, indicating subfunctionalization through regulatory divergence rather than coding‐sequence alteration (Zhou et al. 2016). Low‐temperature‐induced anthocyanin accumulation during postharvest storage is preceded by active DNA demethylation at biosynthetic loci—including *PpPAL1/2*, *PpC4H*, *PpF3H*, *PpDFR*, and *PpANS*—mediated by DNA demethylases and methyltransferases, establishing a temperature–epigenetic axis that links chilling exposure to pigment production (Zhu et al. 2020). The B‐box protein PpBBX32 and the C2H2 zinc‐finger factor PpZAT5 further modulate this response in a tissue‐specific manner, with PpBBX32 activating *PpMYB10.1* and structural genes under chilling conditions (Huang et al. 2024).

Sweet cherry ( *P. avium* ) presents a contrasting pattern in which anthocyanin accumulation is strictly ripening‐dependent. The R2R3‐MYB factor PavMYB10.1 serves as the master regulator of fruit skin color; its overexpression in tobacco induces ectopic anthocyanin production, confirming functional conservation across species (Jin et al. 2016). Light‐induced pigmentation is amplified by PavBBX6 and PavBBX9, which directly bind G‐box elements in the *PavUFGT* promoter and simultaneously activate the ABA biosynthetic gene *PavNCED1*, establishing a positive feedback loop that sustains pigment accumulation during maturation (Wang et al. 2023).

In Japanese plum ( *Prunus salicina* ), postharvest flesh reddening is regulated by the *PsMYB10.1* allele, which is transcriptionally activated at 20°C and upregulates *PsANS*, *PsUFGT*, and *PsGST* to promote coloration; conversely, 30°C suppresses its activity and induces the repressor PsMYB18, which attenuates pigment synthesis (Fang et al. 2021). Cold storage further enhances this process through the PsERF1B–PsMYB10.1–PsbHLH3 module, in which the ethylene‐responsive factor PsERF1B interacts with PsMYB10.1 and PsbHLH3 to form a cold‐specific regulatory complex that boosts transcriptional output on downstream structural genes (Xu et al. 2023).

### 6.5 Cross‐Genera Synthesis: Conserved Modules and Divergent Innovations

Although regulatory architectures exhibit genus‐specific elaborations, several fundamental principles are conserved across Rosaceae. The MBW complex operates as the universal transcriptional hub, with bHLH3/33 and WDR proteins serving as obligate scaffold partners. Activator and repressor MYBs engage in competitive sequestration of shared bHLH factors, while post‐translational modifications—including phosphorylation and ubiquitination—dynamically modulate complex stability and transcriptional output. Table 2 summarizes the key evolutionary innovations that distinguish each genus, highlighting divergence in *MYB10* copy number, environmental responsiveness, epigenetic modulation, hormonal integration, and corresponding breeding priorities (Table 2).

**TABLE 2:** Cross‐genera comparison of anthocyanin regulatory architectures in Rosaceae.

Feature	*Malus*	*Pyrus*	*Fragaria*	*Prunus*
MYB10 copy number	3 (tandem duplication)	2 (divergent paralogs)	2–4 (polyploidy‐dependent)	1 (single locus)
Dominant environmental trigger	Light	Temperature	Light + auxin depletion	Chilling requirement
Key epigenetic mechanism	DNA demethylation	Competitive repressor induction	Histone demethylation	CBF‐mediated chromatin remodeling
Hormone crosstalk	Ethylene/ABA	Ethylene (repressive)	Auxin‐independent	ABA/dormancy hormones
Breeding target	Light‐stable color	Heat tolerance	Uniform epidermal pigmentation	Dormancy‐coupled ripening

*Note:* Comparative summary of genus‐specific innovations in anthocyanin regulatory networks across four major Rosaceae fruit genera. Features highlight evolutionary divergence in MYB10 locus architecture, environmental responsiveness, epigenetic modulation, hormonal integration, and corresponding breeding priorities.

**FIGURE 4:**
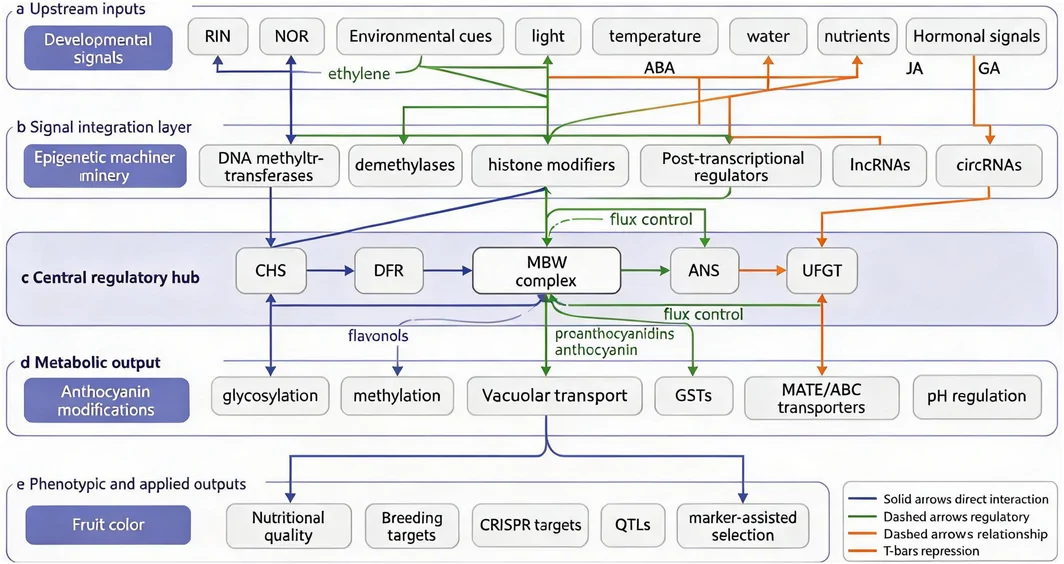
Integrated regulatory network for Rosaceae fruit coloration. Five‐tier hierarchy: (a) upstream inputs (developmental, environmental, hormonal); (b) signal integration (epigenetic machinery, miRNAs, lncRNAs, circRNAs); (c) central hub (MBW complex, structural genes *CHS/DFR/ANS/UFGT*, flux control at flavonol/proanthocyanidin branch points); (d) metabolic output (glycosylation, methylation, acylation, vacuolar transport, pH regulation); (e) phenotypic and applied outputs (fruit color, nutritional quality, QTLs, CRISPR targets, marker‐assisted selection). Solid arrows, direct interactions; dashed arrows, regulatory relationships; green dashed arrows, metabolic flux control.

This comparative framework, supported by a newly added cross‐genera summary figure (Figure 4), provides an integrated reference for understanding how shared molecular toolkits have been repurposed through evolution and domestication to generate the diverse coloration patterns observed across Rosaceae fruits.

## 7 Applications in Breeding and Biotechnology

The mechanistic understanding of anthocyanin regulation in Rosaceae provides a robust foundation for targeted crop improvement. This section bridges fundamental discoveries to practical applications, highlighting how molecular markers, genome editing, synthetic biology, and multi‐omics platforms are accelerating the development of cultivars with optimized coloration, nutritional quality, and environmental resilience.

### 7.1 Molecular Marker‐Assisted Selection

Functional polymorphisms in key regulatory and structural genes have been exploited as diagnostic markers for anthocyanin‐associated traits. In apple, a mini‐satellite insertion in the *MdMYB10* promoter (R6:R1 ratio) serves as a robust marker for red peel coloration, enabling early seedling selection without phenotypic evaluation (Espley et al. 2009). In sweet cherry, a large‐fragment deletion encompassing *PavMYB10.1* underlies the yellow‐fruited phenotype; loss of this allele abolishes anthocyanin synthesis in the skin. The deletion variant has been developed into a PCR‐based diagnostic marker that distinguishes dark‐red, blush, and yellow cultivars, enabling efficient screening of breeding populations for desired color classes (Liu, Qi, et al. 2023). In peach, quantitative trait loci (QTL) mapping has linked a C‐repeat binding factor (CBF) motif variant in the *PpMYB10.1* promoter to chilling requirement and dormancy‐associated pigmentation, facilitating the selection of low‐chill cultivars with consistent fruit coloration (Tuan et al. 2015). These examples demonstrate that regulatory polymorphisms, rather than coding sequence changes, often underlie phenotypic variation and constitute high‐value targets for marker deployment.

### 7.2 CRISPR‐Based Genome Editing

CRISPR (clustered regularly interspaced short palindromic repeats)‐Cas9 technology has been established in Rosaceae fruit crops, although its direct application to anthocyanin pathway genes remains at the proof‐of‐concept stage. In strawberry, CRISPR‐mediated knockout of *FvMAPK3* confirmed that this kinase negatively regulates anthocyanin accumulation by phosphorylating FvMYB10 and promoting FvCHS1 degradation; loss‐of‐function mutants maintained coloration under low‐temperature stress, highlighting the potential of editing signaling nodes to stabilize pigment production under adverse conditions (Mao et al. 2022). In apple, CRISPR‐Cas9 has been successfully deployed to target genes involved in fruit quality and stress responses, establishing the technical framework for future editing of anthocyanin regulators (Zhang, Feng, et al. 2023). These studies illustrate that editing regulatory nodes—rather than structural genes—may offer a strategy to modulate anthocyanin output while minimizing pleiotropic effects.

### 7.3 Synthetic Biology and Metabolic Engineering

Heterologous expression platforms provide complementary routes to anthocyanin production and pathway optimization. The first microbial synthesis of plant‐specific anthocyanins was achieved in *Escherichia coli* by introducing a four‐step pathway comprising F3H and ANS from apple ( *Malus domestica* ), DFR from *Anthurium andraeanum* , and UDP‐glucose: flavonoid 3‐O‐glucosyltransferase (3GT) from *Petunia hybrida* , enabling the conversion of naringenin and eriodictyol to pelargonidin 3‐O‐glucoside and cyanidin 3‐O‐glucoside (Yan et al. 2005). Subsequent metabolic engineering strategies—including precursor supply enhancement, enzyme fusion, and pathway balancing—have elevated titers to 350–439 mg/L cyanidin 3‐O‐glucoside in *E. coli* (Lim et al. 2015). In planta, transient expression systems in Nicotiana benthamiana have been optimized to prototype MBW combinations; co‐expression of Chinese pear ( *Pyrus pyrifolia* ) PpMYB114 with its cognate bHLH partner PpbHLH3 strongly activated core anthocyanin biosynthetic structural genes and triggered massive cyanidin glycoside accumulation in leaf tissue, demonstrating that Rosaceae‐derived TF modules can functionally drive pigment synthesis in heterologous hosts (Ni et al. 2021).

### 7.4 Postharvest Quality Management

Molecular insights into light and temperature signaling inform postharvest technologies that preserve or enhance fruit coloration. In apple, postharvest UV‐B irradiation effectively recovers red pigmentation in poorly colored fruit by upregulating anthocyanin biosynthetic genes; however, excessive UV‐B intensity (> 5 W m^−2^ s^−1^) triggers lignin hyperaccumulation, which compromises skin quality, indicating that dosage optimization is critical (Duan and Eom 2023). In Chinese sand pear ( *Pyrus pyrifolia* ), postharvest UV‐B/visible irradiation at 27°C promotes rapid anthocyanin accumulation, whereas low temperature (17°C) attenuates the response, revealing a positive temperature–light interaction distinct from apple (Sun et al. 2014). In strawberry, red‐light irradiation during storage specifically elevates pelargonidin‐based anthocyanins through an ABA‐dependent pathway, providing a spectral strategy to enhance postharvest coloration without accelerating senescence (Wang, Luo, et al. 2022).

### 7.5 Multi‐Omics Integration in Precision Breeding

The integration of genomics, transcriptomics, and epigenomics is reshaping breeding pipelines for anthocyanin‐related traits. In apple, combined methylome and transcriptome analyses have revealed that ethylene‐mediated hypomethylation at the *MdMYB10* promoter and hypermethylation at *MdbHLH74* coordinately shift the regulatory balance toward anthocyanin accumulation during ripening, demonstrating how epigenomic data can explain genotype–phenotype relationships (Li et al. 2019). In sweet cherry, QTL mapping and association studies have identified *PavMYB10.1* as the major locus controlling skin color, with allelic variation (including a large‐fragment deletion causing yellow fruit) providing diagnostic markers for high‐pigment germplasm selection (Jin et al. 2016; Li et al. 2019; Sooriyapathirana et al. 2010). These multi‐omics platforms enable the construction of predictive models that link genetic variation, epigenetic states, and environmental inputs to anthocyanin accumulation, facilitating genotype–environment optimization in breeding programs.

## 8 Future Perspectives

The study of anthocyanin metabolism in Rosaceae has transitioned from descriptive physiology to mechanistic systems biology (Zhang et al. 2025). Future progress will depend on moving beyond static gene catalogs toward dynamic, predictive models of regulatory network behavior. Several interconnected avenues merit priority attention.

First, multi‐omics integration must advance from correlation to causation. While genome‐wide association and transcriptomic analyses have identified numerous candidate regulators, functional validation in perennial Rosaceae crops remains a bottleneck. Integrated proteogenomic and single‐cell transcriptomic profiling will resolve cell‐type‐specific regulatory architectures. In strawberry, multi‐omics analysis has revealed a lncRNA–miRNA–mRNA regulatory network in which the lncRNA FRILAIR functions as a noncanonical target mimic of miR397 during fruit ripening, competitively reducing miR397‐mediated repression of *LAC11a* and thereby promoting anthocyanin biosynthetic gene expression (Tang et al. 2021). This framework establishes a template for similar network reconstructions across the family. Future studies should leverage such cross‐omic approaches to map dynamic regulatory edges rather than merely catalog node genes.

Second, the genetic and epigenetic basis of species‐specific coloration requires comparative evolutionary interrogation. Research has concentrated disproportionately on apple and strawberry, leaving substantial gaps for peach, pear, cherry, and raspberry. A testable hypothesis is that divergent promoter methylation patterns of *MYB10* homologs underlie genus‐specific temperature sensitivity in anthocyanin accumulation. In peach, low‐temperature‐induced anthocyanin accumulation during postharvest storage is preceded by active DNA demethylation at multiple biosynthetic loci, including *PpPAL1/2*, *PpC4H*, *PpF3H*, *PpDFR*, and *PpANS*, establishing a temperature–epigenetic axis (Zhu et al. 2020). Comparative epigenomics across Rosaceae genera, coupled with CRISPR‐mediated epigenome editing to alter methylation status at *MYB10* promoters, will reveal whether epigenetic divergence is a general mechanism underlying the adaptive radiation of pigmentation traits.

Third, environmental signal integration remains mechanistically opaque. Current studies typically examine single factors (light, temperature, or hormones) in isolation. Future research must decipher how plants perceive and integrate compound stresses—such as combined heat and drought—through convergent hormonal and secondary messenger networks to modulate the core MBW transcriptional machinery. In apple, the GATA transcription factor MdGATA15 functions as a central thermosensor that represses anthocyanin accumulation under heat stress by directly binding to the promoters of *MdMYB11*, *MdANS*, and *MdGSTF12* while activating the repressor *MdMYB308* (Fang et al. 2025). Environmental perturbation combined with temporal multi‐omics offers a promising route to map these signal transduction cascades in real time.

Finally, translating mechanistic knowledge into predictive genetic design represents the ultimate frontier. Although CRISPR‐mediated editing of *FvMAPK3* in strawberry has demonstrated the feasibility of stabilizing anthocyanin accumulation under low‐temperature stress (Mao et al. 2022), future efforts must prioritize multiplexed editing of entire regulatory networks to circumvent pleiotropic effects. Synthetic biology should expand beyond microbial pathway reconstruction to the rational design of environment‐responsive transcriptional circuits in planta. Recent advances in Rosaceae provide a roadmap: in apple, the identification of a functional InDel in the *MdWRKY10* promoter that quantitatively modulates flesh redness offers a target for precision editing (Wang et al. 2024), while in pear, the elucidation of the PpERF9–PpRAP2.4–PpMYB114 repressive module provides a template for engineering synthetic hormone‐responsive switches (Ni et al. 2023). In sweet cherry, the characterization of light‐activated PavBBX6/9 proteins that establish a positive ABA feedback loop suggests strategies for constructing photoreceptor‐linked transcriptional amplifiers (Wang et al. 2023). The convergence of gene editing, epigenetic modulation, and genomic selection will ultimately enable climate‐adaptive breeding of Rosaceae cultivars with optimized coloration and nutritional quality.

## Author Contributions

**Wanjia Tang:** conceptualization, writing – original draft, visualization, software. **Xiaoqing Yuan:** validation, methodology, formal analysis, investigation. **Can Hu:** funding acquisition, writing – review and editing, supervision, validation, project administration, resources. **Menghua Luo:** investigation, formal analysis, conceptualization, methodology, visualization, writing – original draft, data curation.

## Funding

The study was partially funded by the Natural Science Foundation of Sichuan Province, China (2025ZNSFSC1100).

## Conflicts of Interest

The authors declare no conflicts of interest.

## Data Availability

The authors have nothing to report.

## References

[fsn372269-bib-0001] Achnine, L. , E. B. Blancaflor , S. Rasmussen , and R. A. Dixon . 2004. “Colocalization of L‐Phenylalanine Ammonia‐Lyase and Cinnamate 4‐Hydroxylase for Metabolic Channeling in Phenylpropanoid Biosynthesis.” Plant Cell 16, no. 11: 3098–3109. 10.1105/tpc.104.024406. 15472080 PMC527201

[fsn372269-bib-0002] Alabd, A. , M. Ahmad , X. Zhang , et al. 2022. “Light‐Responsive Transcription Factor PpWRKY44 Induces Anthocyanin Accumulation by Regulating PpMYB10 Expression in Pear.” Horticulture Research 9: uhac199. 10.1093/hr/uhac199. 37180030 PMC10167416

[fsn372269-bib-0003] An, J. , X. Zhang , S. Bi , C. You , X. Wang , and Y. Hao . 2020. “The ERF Transcription Factor MdERF38 Promotes Drought Stress‐Induced Anthocyanin Biosynthesis in Apple.” Plant Journal 101, no. 3: 573–589. 10.1111/tpj.14555. 31571281

[fsn372269-bib-0004] An, J. , X. Zhang , Y. Liu , X. Wang , C. You , and Y. Hao . 2021. “ABI5 Regulates ABA‐Induced Anthocyanin Biosynthesis by Modulating the MYB1‐bHLH3 Complex in Apple.” Journal of Experimental Botany 72, no. 4: 1460–1472. 10.1093/jxb/eraa525. 33159793

[fsn372269-bib-0005] An, J. , X. Zhang , C. You , S. Bi , X. Wang , and Y. Hao . 2019. “MdWRKY40 Promotes Wounding‐Induced Anthocyanin Biosynthesis in Association With MdMYB1 and Undergoes MdBT2‐Mediated Degradation.” New Phytologist 224, no. 1: 380–395. 10.1111/nph.16008. 31225908

[fsn372269-bib-0006] Ao, P. , Y. Ma , K. Luo , et al. 2025. “Transcriptomic and Metabolic Profiles of Apple Peels of Different Colors.” Plants 14, no. 21: 3304. 10.3390/plants14213304. 41225855 PMC12610381

[fsn372269-bib-0007] Bai, S. , R. Tao , Y. Tang , et al. 2019. “BBX16, a B‐Box Protein, Positively Regulates Light‐Induced Anthocyanin Accumulation by Activating MYB10 in Red Pear.” Plant Biotechnology Journal 17, no. 10: 1985–1997. 10.1111/pbi.13114. 30963689 PMC6737026

[fsn372269-bib-0008] Bhosale, S. S. , D. Granato , and N. Mohammadi . 2025. “Purification and Characterization of Anthocyanin From Irish Wild Blackberry: Impact on Color, Composition, and Antioxidant Capacity.” Journal of Food Measurement and Characterization 19, no. 4: 2730–2738. 10.1007/s11694-025-03142-8.

[fsn372269-bib-0009] Bouillon, P. , E. Belin , A. Fanciullino , et al. 2025. “Fade Into You: Genetic Control of Pigmentation Patterns in Red‐Flesh Apple (*Malus* × *Domestica*).” Frontiers in Plant Science 15: 1462545. 10.3389/fpls.2024.1462545. 39872201 PMC11770013

[fsn372269-bib-0010] Buhrman, K. , J. Aravena‐Calvo , C. R. Zaulich , K. Hinz , and T. Laursen . 2022. “Anthocyanic Vacuolar Inclusions: From Biosynthesis to Storage and Possible Applications.” Frontiers in Chemistry 10: 913324. 10.3389/fchem.2022.913324. 35836677 PMC9273883

[fsn372269-bib-0011] Cai, H. , S. Zhang , Y. Zhang , Y. Zhou , and L. Li . 2019. “Epigenetic Regulation of Anthocyanin Biosynthesis by an Antagonistic Interaction Between H2A.Z and H3K4me3.” New Phytologist 221, no. 1: 295–308. 10.1111/nph.15306. 29959895

[fsn372269-bib-0012] Cappellini, F. , A. Marinelli , M. Toccaceli , C. Tonelli , and K. Petroni . 2021. “Anthocyanins: From Mechanisms of Regulation in Plants to Health Benefits in Foods.” Frontiers in Plant Science 12: 748049. 10.3389/fpls.2021.748049. 34777426 PMC8580863

[fsn372269-bib-0013] Castillejo, C. , V. Waurich , H. Wagner , et al. 2020. “Allelic Ariation of MYB10 Is the Major Force Controlling Natural Variation in Skin and Flesh Color in Strawberry (*Fragaria* spp.) Fruit.” Plant Cell 32, no. 12: 3723–3749. 10.1105/tpc.20.00474. 33004617 PMC7721342

[fsn372269-bib-0014] Chagné, D. , K. Lin‐Wang , R. V. Espley , et al. 2013. “An Ancient Duplication of Apple MYB Transcription Factors Is Responsible for Novel Red Fruit‐Flesh Phenotypes.” Plant Physiology 161, no. 1: 225–239. 10.1104/pp.112.206771. 23096157 PMC3532254

[fsn372269-bib-0015] Chen, L. , X. Tian , Y. Feng , et al. 2024. “The Genome‐Wide Identification of the *Dihydroflavonol 4‐Reductase* (*DFR*) Gene Family and Its Expression Analysis in Different Fruit Coloring Stages of Strawberry.” International Journal of Molecular Sciences 25, no. 18: 9911. 10.3390/ijms25189911. 39337399 PMC11432397

[fsn372269-bib-0016] Cheng, J. , G. Wei , H. Zhou , et al. 2014. “Unraveling the Mechanism Underlying the Glycosylation and Methylation of Anthocyanins in Peach.” Plant Physiology 166, no. 2: 1044–1058. 10.1104/pp.114.246876. 25106821 PMC4213075

[fsn372269-bib-0017] Cong, L. , Y. Qu , G. Sha , et al. 2021. “PbWRKY75 Promotes Anthocyanin Synthesis by Activating PbDFR, PbUFGT, and PbMYB10b in Pear.” Physiologia Plantarum 173, no. 4: 1841–1849. 10.1111/ppl.13525. 34418106

[fsn372269-bib-0018] Dai, J. , Z. Xu , Z. Fang , et al. 2024. “NAC Transcription Factor PpNAP4 Promotes Chlorophyll Degradation and Anthocyanin Synthesis in the Skin of Peach Fruit.” Journal of Agricultural and Food Chemistry 72, no. 36: 19826–19837. 10.1021/acs.jafc.4c03924. 39213503

[fsn372269-bib-0019] Du, M. , Y. Wang , X. Zhang , et al. 2025. “Integrated Metabolomic and Transcriptomic Analyses Elucidate Anthocyanin‐Mediated Flesh Coloration Mechanisms in Red‐Fleshed Pear.” Frontiers in Plant Science 16: 1670229. 10.3389/fpls.2025.1670229. 41245451 PMC12615504

[fsn372269-bib-0020] Duan, S. , and S. H. Eom . 2023. “Regulation of Anthocyanin and Lignin Contents in Postharvest ‘Fuji’ Apple Irradiated With UV‐B.” Scientia Horticulturae 312: 112428. 10.1016/j.scienta.2023.112428.

[fsn372269-bib-0021] Er An, S. , M. Müller , L. Reuter , R. Carle , and J. Müller‐Maatsch . 2022. “Co‐Pigmentation of Strawberry Anthocyanins With Phenolic Compounds From Rooibos.” Food Chemistry 4: 100097. 10.1016/j.fochms.2022.100097. 35769401 PMC9235051

[fsn372269-bib-0022] Espley, R. V. , C. Brendolise , D. Chagné , et al. 2009. “Multiple Repeats of a Promoter Segment Causes Transcription Factor Autoregulation in Red Apples.” Plant Cell 21, no. 1: 168–183. 10.1105/tpc.108.059329. 19151225 PMC2648084

[fsn372269-bib-0023] Espley, R. V. , R. P. Hellens , J. Putterill , D. E. Stevenson , S. Kutty‐Amma , and A. C. Allan . 2007. “Red Colouration in Apple Fruit Is due to the Activity of the MYB Transcription Factor, MdMYB10.” Plant Journal 49, no. 3: 414–427. 10.1111/j.1365-313X.2006.02964.x. PMC1865000 17181777

[fsn372269-bib-0024] Fang, H. , Y. Dong , X. Yue , et al. 2019. “MdCOL4 Interaction Mediates Crosstalk Between UV‐B and High Temperature to Control Fruit Coloration in Apple.” Plant & Cell Physiology 60, no. 5: 1055–1066. 10.1093/pcp/pcz023. 30715487

[fsn372269-bib-0025] Fang, H. , Y. Dong , X. Yue , et al. 2019. “The B‐Box Zinc Finger Protein MdBBX20 Integrates Anthocyanin Accumulation in Response to Ultraviolet Radiation and Low Temperature.” Plant, Cell & Environment 42, no. 7: 2090–2104. 10.1111/pce.13552. 30919454

[fsn372269-bib-0026] Fang, X. , L. Zhang , L. Shangguan , and L. Wang . 2023. “MdMYB110a, Directly and Indirectly, Activates the Structural Genes for the ALA‐Induced Accumulation of Anthocyanin in Apple.” Plant Science 326: 111511. 10.1016/j.plantsci.2022.111511. 36377142

[fsn372269-bib-0027] Fang, Y. , H. Ma , Z. Wu , et al. 2025. “High Temperature Induces MdGATA15 to Suppress Anthocyanin Accumulation in Apple Peels.” Plant Journal 123, no. 2: e70381. 10.1111/tpj.70381. 40714847

[fsn372269-bib-0028] Fang, Z. , K. Lin‐Wang , C. Jiang , et al. 2021. “Postharvest Temperature and Light Treatments Induce Anthocyanin Accumulation in Peel of ‘Akihime’ Plum ( *Prunus salicina* Lindl.) via Transcription Factor PsMYB10.1.” Postharvest Biology and Technology 8: 66. 10.1038/s41438-021-00634-8.

[fsn372269-bib-0029] Fang, Z. , Y. Wang , and C. Wang . 2022. “Metabolomic and Transcriptomic Analyses Provide Insights Into Temperature and Light Regulated Anthocyanin Accumulation in Flesh of ‘Furongli’ Plum ( *Prunus salicina* Lindl.).” Plant Physiology and Biochemistry 177: 107978. 10.1016/j.plaphy.2022.107978.

[fsn372269-bib-0030] Feng, S. , X. Chen , C. Zhang , et al. 2008. “Relationship Between Anthocyanin Biosynthesis and Related Enzymes Activity in *Pyrus pyrifolia* Mantianhong and Its Bud Sports Aoguan.” Agricultural Sciences in China 7, no. 11: 1318–1323. 10.1016/S1671-2927(08)60180-7.

[fsn372269-bib-0031] Feng, Y. , S. Yang , W. Li , J. Mao , B. Chen , and Z. Ma . 2023. “Genome‐Wide Identification and Expression Analysis of ANS Family in Strawberry Fruits at Different Coloring Stages.” International Journal of Molecular Sciences 24, no. 16: 12554. 10.3390/ijms241612554. 37628740 PMC10454780

[fsn372269-bib-0032] Frank, S. , M. Keck , M. Sagasser , K. Niehaus , B. Weisshaar , and R. Stracke . 2011. “Two Differentially Expressed MATE Factor Genes From Apple Complement the Arabidopsis Transparent Testa12 Mutant.” Plant Biology 13, no. 1: 42–50. 10.1111/j.1438-8677.2010.00350.x. 21143724

[fsn372269-bib-0033] Gomez, C. , G. Conejero , L. Torregrosa , V. Cheynier , N. Terrier , and A. Ageorges . 2011. “In Vivo Grapevine Anthocyanin Transport Involves Vesicle‐Mediated Trafficking and the Contribution of anthoMATE Transporters and GST.” Plant Journal 67, no. 6: 960–970. 10.1111/j.1365-313X.2011.04648.x. 21605207

[fsn372269-bib-0034] Grünig, N. , J. M. Horz , and B. Pucker . 2025. “Diversity and Ecological Functions of Anthocyanins.” BMC Plant Biology 26, no. 1: 146. 10.1186/s12870-025-08006-3. 41436926 PMC12837240

[fsn372269-bib-0035] Henry‐Kirk, R. A. , B. Plunkett , M. Hall , et al. 2018. “Solar UV Light Regulates Flavonoid Metabolism in Apple ( *Malus domestica* ).” Plant, Cell & Environment 41, no. 3: 675–688. 10.1111/pce.13125. 29315644

[fsn372269-bib-0036] Hu, D. , C. Sun , Q. Ma , C. You , L. Cheng , and Y. Hao . 2016. “MdMYB1 Regulates Anthocyanin and Malate Accumulation by Directly Facilitating Their Transport Into Vacuoles in Apples.” Plant Physiology 170, no. 3: 1315–1330. 10.1104/pp.15.01333. 26637549 PMC4775115

[fsn372269-bib-0037] Hu, Y. , Z. Gong , Y. Yan , et al. 2024. “ChBBX6 and ChBBX18 Are Positive Regulators of Anthocyanins Biosynthesis and Carotenoids Degradation in Cerasus Humilis.” International Journal of Biological Macromolecules 282, no. 4: 137195. 10.1016/j.ijbiomac.2024.137195. 39489264

[fsn372269-bib-0038] Huang, D. , L. Xue , Y. Lu , et al. 2024. “PpBBX32 and PpZAT5 Modulate Temperature‐Dependent and Tissue‐Specific Anthocyanin Accumulation in Peach Fruit.” Horticulture Research 11, no. 10: uhae212. 10.1093/hr/uhae212. 39385999 PMC11462610

[fsn372269-bib-0039] Huang, Y. , W. Li , S. Jiao , J. Huang , B. Chen , and J. Kormanec . 2023. “MdMYB66 Is Associated With Anthocyanin Biosynthesis via the Activation of the MdF3H Promoter in the Fruit Skin of an Apple Bud Mutant.” International Journal of Molecular Sciences 24, no. 23: 16871. 10.3390/ijms242316871. 38069191 PMC10706036

[fsn372269-bib-0040] Iqbal, M. , L. Zhang , Y. Yin , Z. Yang , J. Zhang , and L. Wang . 2026. “MdBBX47, a B‐Box Transcription Factor Directly and Indirectly Regulates ALA‐Induced Anthocyanin Accumulation in Apple.” BMC Plant Biology 26, no. 1: 1038. 10.1186/s12870-026-08838-7. 42056891 PMC13270602

[fsn372269-bib-0041] Jaakola, L. 2013. “New Insights Into the Regulation of Anthocyanin Biosynthesis in Fruits.” Trends in Plant Science 18, no. 9: 477–483. 10.1016/j.tplants.2013.06.003. 23870661

[fsn372269-bib-0042] Jia, H. , Y. Chai , C. Li , et al. 2011. “Abscisic Acid Plays an Important Role in the Regulation of Strawberry Fruit Ripening.” Plant Physiology 157, no. 1: 188–199. 10.1104/pp.111.177311. 21734113 PMC3165869

[fsn372269-bib-0043] Jiang, L. , M. Yue , Y. Liu , et al. 2023. “A Novel R2R3‐MYB Transcription Factor FaMYB5 Positively Regulates Anthocyanin and Proanthocyanidin Biosynthesis in Cultivated Strawberries (*Fragaria* × *Ananassa*).” Plant Biotechnology Journal 21, no. 6: 1140–1158. 10.1111/pbi.14024. 36752420 PMC10214752

[fsn372269-bib-0044] Jiang, S. , M. Chen , N. He , et al. 2019. “MdGSTF6, Activated by MdMYB1, Plays an Essential Role in Anthocyanin Accumulation in Apple.” Horticulture Research 6: 40. 10.1038/s41438-019-0118-6. 30854214 PMC6395711

[fsn372269-bib-0045] Jiang, S. , N. Wang , M. Chen , et al. 2020. “Methylation of MdMYB1 Locus Mediated by RdDM Pathway Regulates Anthocyanin Biosynthesis in Apple.” Plant Biotechnology Journal 18, no. 8: 1736–1748. 10.1111/pbi.13337. 31930634 PMC7336386

[fsn372269-bib-0046] Jin, W. , H. Wang , M. Li , et al. 2016. “The R2R3 MYB Transcription Factor PavMYB10.1 Involves in Anthocyanin Biosynthesis and Determines Fruit Skin Colour in Sweet Cherry ( *Prunus avium* L.).” Plant Biotechnology Journal 14, no. 11: 2120–2133. 10.1111/pbi.12568. 27107393 PMC5095807

[fsn372269-bib-0047] Jones, J. A. , V. R. Vernacchio , S. M. Collins , et al. 2017. “Complete Biosynthesis of Anthocyanins Using *E. coli* Polycultures.” MBio 8, no. 3: e00621‐17. 10.1128/mBio.00621-17. 28588129 PMC5461408

[fsn372269-bib-0048] Kallam, K. , I. Appelhagen , J. Luo , et al. 2017. “Aromatic Decoration Determines the Formation of Anthocyanic Vacuolar Inclusions.” Current Biology 27, no. 7: 945–957. 10.1016/j.cub.2017.02.027. 28318977 PMC5387179

[fsn372269-bib-0049] Khoo, H. E. , A. Azlan , S. T. Tang , and S. M. Lim . 2017. “Anthocyanidins and Anthocyanins: Colored Pigments as Food, Pharmaceutical Ingredients, and the Potential Health Benefits.” Food & Nutrition Research 61, no. 1: 1361779. 10.1080/16546628.2017.1361779. 28970777 PMC5613902

[fsn372269-bib-0050] Koes, R. , W. Verweij , and F. Quattrocchio . 2005. “Flavonoids: A Colorful Model for the Regulation and Evolution of Biochemical Pathways.” Trends in Plant Science 10, no. 5: 236–242. 10.1016/j.tplants.2005.03.002. 15882656

[fsn372269-bib-0051] Kong, Y. , X. Wang , Z. Wu , Y. Li , F. Xu , and F. Xie . 2023. “Enzymatic Acylation of Black Rice Anthocyanins and Evaluation of Antioxidant Capacity and Stability of Their Derivatives.” Food 12, no. 24: 4505. 10.3390/foods12244505. PMC10743184 38137310

[fsn372269-bib-0052] Kou, X. , Z. Zhao , X. Xu , C. Li , J. Wu , and S. Zhang . 2024. “Identification and Expression Analysis of ATP‐Binding Cassette (ABC) Transporters Revealed Its Role in Regulating Stress Response in Pear (*Pyrus bretchneideri*).” BMC Genomics 25, no. 1: 169. 10.1186/s12864-024-10063-1. 38347517 PMC10863237

[fsn372269-bib-0053] Kou, Y. , J. Ren , Y. Ma , et al. 2023. “Effects of Exogenous Substances Treatment on Fruit Quality and Pericarp Anthocyanin Metabolism of Peach.” Agronomy 13, no. 6: 1489. 10.3390/agronomy13061489.

[fsn372269-bib-0054] Lei, T. , J. Huang , H. Ruan , et al. 2023. “Competition Between FLS and DFR Regulates the Distribution of Flavonols and Proanthocyanidins in Rubus Chingii Hu.” Frontiers in Plant Science 14: 1134993. 10.3389/fpls.2023.1134993. 36968391 PMC10031046

[fsn372269-bib-0055] Li, C. , H. Jia , Y. Chai , and Y. Shen . 2011. “Abscisic Acid Perception and Signaling Transduction in Strawberry: A Model for Non‐Climacteric Fruit Ripening.” Plant Signaling & Behavior 6, no. 12: 1950–1953. 10.4161/psb.6.12.18024. 22095148 PMC3337185

[fsn372269-bib-0056] Li, C. , J. Wu , K. Hu , et al. 2020. “PyWRKY26 and PybHLH3 Cotargeted the PyMYB114 Promoter to Regulate Anthocyanin Biosynthesis and Transport in Red‐Skinned Pears.” Horticulture Research 7: 37. 10.1038/s41438-020-0254-z. 32194973 PMC7072072

[fsn372269-bib-0057] Li, H. , Z. Liu , X. Wang , Y. Han , C. You , and J. An . 2023. “E3 Ubiquitin Ligases SINA4 and SINA11 Regulate Anthocyanin Biosynthesis by Targeting the IAA29‐ARF5‐1‐ERF3 Module in Apple.” Plant, Cell & Environment 46, no. 12: 3902–3918. 10.1111/pce.14709. 37658649

[fsn372269-bib-0058] Li, W. , J. Gao , Z. Ma , et al. 2023. “Molecular Evolution and Expression Assessment of DFRs in Apple.” Chemical and Biological Technologies in Agriculture 10, no. 1: 98. 10.1186/s40538-023-00470-z.

[fsn372269-bib-0059] Li, W. , J. Mao , S. Yang , et al. 2018. “Anthocyanin Accumulation Correlates With Hormones in the Fruit Skin of ‘Red Delicious’ and Its Four Generation Bud Sport Mutants.” BMC Plant Biology 18, no.1: 363. 10.1186/s12870-018-1595-8. 30563462 PMC6299587

[fsn372269-bib-0060] Li, W. , G. Ning , J. Mao , Z. Guo , Q. Zhou , and B. Chen . 2019. “Whole‐Genome DNA Methylation Patterns and Complex Associations With Gene Expression Associated With Anthocyanin Biosynthesis in Apple Fruit Skin.” Planta 250, no. 6: 1833–1847. 10.1007/s00425-019-03266-4. 31471637

[fsn372269-bib-0061] Lim, C. G. , L. Wong , N. Bhan , et al. 2015. “Development of a Recombinant *Escherichia coli* Strain for Overproduction of 3‐O‐Glycosylated Flavonoids.” Metabolic Engineering 31: 74–81. 10.1016/j.ymben.2015.07.001.26166409

[fsn372269-bib-0062] Lin‐Wang, K. , T. K. Mcghie , M. Wang , et al. 2014. “Engineering the Anthocyanin Regulatory Complex of Strawberry ( *Fragaria vesca* ).” Frontiers in Plant Science 5: 651. 10.3389/fpls.2014.00651. 25477896 PMC4237049

[fsn372269-bib-0063] Liu, C. , X. Qi , L. Song , et al. 2023. “Large‐Fragment Deletion Encompasses the R2R3 MYB Transcription Factor, PavMYB10.1, Causes Yellow Fruits in Sweet Cherry ( *Prunus avium* L.).” Scientia Horticulturae 309: 111648. 10.1016/j.scienta.2022.111648.

[fsn372269-bib-0064] Liu, G. , D. Fu , X. Duan , et al. 2024. “Integrated Metabolome, Transcriptome and Long Non‐Coding RNA Analysis Reveals Potential Molecular Mechanisms of Sweet Cherry Fruit Ripening.” International Journal of Molecular Sciences 25, no. 18: 9860. 10.3390/ijms25189860. 39337346 PMC11432518

[fsn372269-bib-0065] Liu, H. , Q. Shu , K. Lin‐Wang , et al. 2023. “DNA Methylation Reprogramming Provides Insights Into Light‐Induced Anthocyanin Biosynthesis in Red Pear.” Plant Science 326: 111499. 10.1016/j.plantsci.2022.111499. 36265764

[fsn372269-bib-0066] Liu, H. , J. Su , Y. Zhu , et al. 2019. “The Involvement of PybZIPa in Light‐Induced Anthocyanin Accumulation via the Activation of PyUFGT Through Binding to Tandem G‐Boxes in Its Promoter.” Horticulture Research 6: 134. 10.1038/s41438-019-0217-4. 31814987 PMC6885052

[fsn372269-bib-0067] Liu, W. , Z. Mei , L. Yu , et al. 2023. “The ABA‐Induced NAC Transcription Factor MdNAC1 Interacts With a bZIP‐Type Transcription Factor to Promote Anthocyanin Synthesis in Red‐Fleshed Apples.” Horticulture Research 10, no. 5: uhad49. 10.1093/hr/uhad049. PMC10186271 37200839

[fsn372269-bib-0068] Liu, W. , Y. Wang , L. Yu , et al. 2019. “MdWRKY11 Participates in Anthocyanin Accumulation in Red‐Fleshed Apples by Affecting MYB Transcription Factors and the Photoresponse Factor MdHY5.” Journal of Agricultural and Food Chemistry 67, no. 32: 8783–8793. 10.1021/acs.jafc.9b02920. 31310107

[fsn372269-bib-0069] Liu, Y. , F. Che , L. Wang , R. Meng , X. Zhang , and Z. Zhao . 2013. “Fruit Coloration and Anthocyanin Biosynthesis After Bag Removal in Non‐Red and Red Apples (*Malus* × *Domestica* Borkh.).” Molecules 18, no. 2: 1549–1563. 10.3390/molecules18021549. 23353125 PMC6269864

[fsn372269-bib-0070] Liu, Y. , X. Shen , K. Zhao , et al. 2013. “Expression Analysis of Anthocyanin Biosynthetic Genes in Different Colored Sweet Cherries ( *Prunus avium* L.) During Fruit Development.” Journal of Plant Growth Regulation 32, no. 4: 901–907. 10.1007/s00344-013-9355-3.

[fsn372269-bib-0071] Liu, Y. , L. Tang , Y. Wang , et al. 2023. “The Blue Light Signal Transduction Module FaCRY1‐FaCOP1‐FaHY5 Regulates Anthocyanin Accumulation in Cultivated Strawberry.” Frontiers in Plant Science 14: 1144273. 10.3389/fpls.2023.1144273. 37360713 PMC10289005

[fsn372269-bib-0072] Liu, Y. , Y. Ye , Y. Wang , et al. 2022. “B‐Box Transcription Factor FaBBX22 Promotes Light‐Induced Anthocyanin Accumulation in Strawberry (*Fragaria* × *Ananassa*).” International Journal of Molecular Sciences 23, no. 14: 7757. 10.3390/ijms23147757. 35887106 PMC9316111

[fsn372269-bib-0073] Liu, Z. , H. Ma , S. Jung , D. Main , and L. Guo . 2020. “Developmental Mechanisms of Fleshy Fruit Diversity in Rosaceae.” Annual Review of Plant Biology 71: 547–573. 10.1146/annurev-arplant-111119-021700. 32442388

[fsn372269-bib-0074] Lunkenbein, S. , H. Coiner , C. H. R. de Vos , et al. 2006. “Molecular Characterization of a Stable Antisense Chalcone Synthase Phenotype in Strawberry (*Fragaria* × *Ananassa*).” Journal of Agricultural and Food Chemistry 54, no. 6: 2145–2153. 10.1021/jf052574z. 16536589

[fsn372269-bib-0075] Luo, H. , C. Dai , Y. Li , J. Feng , Z. Liu , and C. Kang . 2018. “Reduced Anthocyanins in Petioles Codes for a GST Anthocyanin Transporter That Is Essential for the Foliage and Fruit Coloration in Strawberry.” Journal of Experimental Botany 69, no. 10: 2595–2608. 10.1093/jxb/ery096. 29538703 PMC5920330

[fsn372269-bib-0076] Ma, D. , H. Tang , M. Reichelt , et al. 2021. “Poplar MYB117 Promotes Anthocyanin Synthesis and Enhances Flavonoid B‐Ring Hydroxylation by Up‐Regulating the Flavonoid 3 Hydroxylase Gene.” Journal of Experimental Botany 72, no. 10: 3864–3880. 10.1093/jxb/erab116. 33711094

[fsn372269-bib-0077] Ma, H. , T. Yang , Y. Li , et al. 2021. “The Long Noncoding RNA MdLNC499 Bridges MdWRKY1 and MdERF109 Function to Regulate Early‐Stage Light‐Induced Anthocyanin Accumulation in Apple Fruit.” Plant Cell 33, no. 10: 3309–3330. 10.1093/plcell/koab188. 34270784 PMC8505877

[fsn372269-bib-0078] Mao, W. , Y. Han , Y. Chen , et al. 2022. “Low Temperature Inhibits Anthocyanin Accumulation in Strawberry Fruit by Activating FvMAPK3‐Induced Phosphorylation of FvMYB10 and Degradation of Chalcone Synthase 1.” Plant Cell 34, no. 4: 1226–1249. 10.1093/plcell/koac006. 35018459 PMC8972286

[fsn372269-bib-0079] Martínez‐Ferri, E. , T. Y. Forbes‐Hernandez , L. Cervantes , C. Soria , M. Battino , and M. T. Ariza . 2023. “Relation Between Strawberry Fruit Redness and Bioactivity: Deciphering the Role of Anthocyanins as Health Promoting Compounds.” Food 13, no. 1: 110. 10.3390/foods13010110. PMC10778386 38201141

[fsn372269-bib-0080] Moghe, G. , L. H. Kruse , M. Petersen , et al. 2023. “BAHD Company: The Ever‐Expanding Roles of the BAHD Acyltransferase Gene Family in Plants.” Annual Review of Plant Biology 74: 165–194. 10.1146/annurev-arplant-062922-050122. 36450296

[fsn372269-bib-0081] Moyer, R. A. , K. E. Hummer , C. E. Finn , B. Frei , and R. E. Wrolstad . 2002. “Anthocyanins, Phenolics, and Antioxidant Capacity in Diverse Small Fruits: Vaccinium, Rubus, and Ribes.” Journal of Agricultural and Food Chemistry 50, no. 3: 519–525. 10.1021/jf011062r. 11804523

[fsn372269-bib-0082] Muhammad, N. , Z. Luo , M. Yang , X. Li , Z. Liu , and M. Liu . 2022. “The Joint Role of the Late Anthocyanin Biosynthetic UFGT‐Encoding Genes in the Flowers and Fruits Coloration of Horticultural Plants.” Scientia Horticulturae 301: 111110. 10.1016/j.scienta.2022.111110.

[fsn372269-bib-0083] Ni, J. , S. Bai , Y. Zhao , et al. 2019. “Ethylene Response Factors Pp4ERF24 and Pp12ERF96 Regulate Blue Light‐Induced Anthocyanin Biosynthesis in ‘Red Zaosu’ Pear Fruits by Interacting With MYB114.” Plant Molecular Biology 99, no. 1–2: 67–78. 10.1007/s11103-018-0802-1. 30539403

[fsn372269-bib-0084] Ni, J. , A. T. Premathilake , Y. Gao , et al. 2021. “Ethylene‐Activated PpERF105 Induces the Expression of the Repressor‐Type R2R3‐MYB Gene PpMYB140 to Inhibit Anthocyanin Biosynthesis in Red Pear Fruit.” Plant Journal 105, no. 1: 167–181. 10.1111/tpj.15049. 33111423

[fsn372269-bib-0085] Ni, J. , S. Wang , W. Yu , et al. 2023. “The Ethylene‐Responsive Transcription Factor PpERF9 Represses PpRAP2.4 and PpMYB114 via Histone Deacetylation to Inhibit Anthocyanin Biosynthesis in Pear.” Plant Cell 35, no. 6: 2271–2292. 10.1093/plcell/koad077. 36916511 PMC10226596

[fsn372269-bib-0086] Nowicka, A. , A. Z. Kucharska , A. Sokó Towska , and I. Fecka . 2019. “Comparison of Polyphenol Content and Antioxidant Capacity of Strawberry Fruit From 90 Cultivars of *Fragaria* × *Ananassa* Duch.” Food Chemistry 270: 32–46. 10.1016/j.foodchem.2018.07.015. 30174053

[fsn372269-bib-0087] Ono, E. , Y. Homma , M. Horikawa , et al. 2010. “Functional Differentiation of the Glycosyltransferases That Contribute to the Chemical Diversity of Bioactive Flavonol Glycosides in Grapevines ( *Vitis vinifera* ).” Plant Cell 22, no. 8: 2856–2871. 10.1105/tpc.110.074625. 20693356 PMC2947185

[fsn372269-bib-0088] Pan, Q. , S. Guo , J. Ding , et al. 2024. “Dynamic Histone Modification Signatures Coordinate Developmental Programs in Strawberry Fruit Ripening.” Horticulture Research 11, no. 8: uhae158. 10.1093/hr/uhae158. 39108587 PMC11298626

[fsn372269-bib-0089] Pei, Y. , W. Tang , Y. Huang , et al. 2025. “The PavMYB.C2‐UFGT Module Contributes to Fruit Coloration via Modulating Anthocyanin Biosynthesis in Sweet Cherry.” PLoS Genetics 21, no. 6: e1011761. 10.1371/journal.pgen.1011761. 40526782 PMC12185008

[fsn372269-bib-0090] Peng, Z. , J. Tian , R. Luo , et al. 2020. “MiR399d and Epigenetic Modification Comodulate Anthocyanin Accumulation in Malus Leaves Suffering From Phosphorus Deficiency.” Plant, Cell & Environment 43, no. 5: 1148–1159. 10.1111/pce.13697. 31833568

[fsn372269-bib-0091] Perotti, M. F. , D. Posé , and C. Martín‐Pizarro . 2023. “Non‐Climacteric Fruit Development and Ripening Regulation: ‘The Phytohormones Show’.” Journal of Experimental Botany 74, no. 20: 6237–6253. 10.1093/jxb/erad271. 37449770 PMC10627154

[fsn372269-bib-0092] Qi, Q. , M. Chu , X. Yu , et al. 2023. “Anthocyanins and Proanthocyanidins: Chemical Structures, Food Sources, Bioactivities, and Product Development.” Food Reviews International 39, no. 7: 4581–4609. 10.1080/87559129.2022.2029479.

[fsn372269-bib-0093] Ramsay, N. A. , and B. J. Glover . 2005. “MYB‐bHLH‐WD40 Protein Complex and the Evolution of Cellular Diversity.” Trends in Plant Science 10, no. 2: 63–70. 10.1016/j.tplants.2004.12.011. 15708343

[fsn372269-bib-0094] Sargent, D. J. , M. Buti , S. Martens , et al. 2025. “A CACTA‐Like Transposon in the Anthocyanidin Synthase 1 (Ans‐1) Gene Is Responsible for Apricot Fruit Colour in the Raspberry ( *Rubus idaeus* ) Cultivar ‘Varnes’.” PLoS One 20, no. 2: e318692. 10.1371/journal.pone.0318692. PMC11790086 39899506

[fsn372269-bib-0095] Shulaev, V. , D. J. Sargent , R. N. Crowhurst , et al. 2011. “The Genome of Woodland Strawberry ( *Fragaria vesca* ).” Nature Genetics 43, no. 2: 109–116. 10.1038/ng.740. 21186353 PMC3326587

[fsn372269-bib-0096] Sooriyapathirana, S. , A. Khan , A. Sebolt , et al. 2010. “QTL Analysis and Candidate Gene Mapping for Skin and Flesh Color in Sweet Cherry Fruit ( *Prunus avium* L.).” Tree Genetics & Genomes 6, no. 6: 821–832. 10.1007/s11295-010-0296-0.

[fsn372269-bib-0097] Sun, H. , F. Cui , Y. Liu , L. Qian , S. Zhu , and Y. Li . 2025. “Integrated Metabolomics and Transcriptomics Unravel the Biosynthesis Mechanism of Anthocyanin in Postharvest Red Raspberry ( *Rubus idaeus* L.).” Frontiers in Plant Science 16: 1549458. 10.3389/fpls.2025.1549458. 40433162 PMC12106431

[fsn372269-bib-0098] Sun, L. , J. Huo , J. Liu , et al. 2023. “Anthocyanins Distribution, Transcriptional Regulation, Epigenetic and Post‐Translational Modification in Fruits.” Food Chemistry 411: 135540. 10.1016/j.foodchem.2023.135540. 36701918

[fsn372269-bib-0099] Sun, Y. , M. Qian , R. Wu , Q. Niu , Y. Teng , and Z. Li . 2014. “Postharvest Pigmentation in Red Chinese Sand Pears ( *Pyrus pyrifolia* Nakai.) in Response to Optimum Light and Temperature.” Postharvest Biology and Technology 91: 64–71. 10.1016/j.postharvbio.2014.01.007.

[fsn372269-bib-0100] Sunil, L. , and N. P. Shetty . 2022. “Biosynthesis and Regulation of Anthocyanin Pathway Genes.” Applied Microbiology and Biotechnology 106, no. 5–6: 1783–1798. 10.1007/s00253-022-11835-z. 35171341

[fsn372269-bib-0101] Tang, Y. , Z. Qu , J. Lei , et al. 2021. “The Long Noncoding RNA FRILAIR Regulates Strawberry Fruit Ripening by Functioning as a Noncanonical Target Mimic.” PLoS Genetics 17, no. 3: e1009461. 10.1371/journal.pgen.1009461. 33739974 PMC8011760

[fsn372269-bib-0102] Telias, A. , K. Lin‐Wang , D. E. Stevenson , et al. 2011. “Apple Skin Patterning Is Associated With Differential Expression of MYB10.” BMC Plant Biology 11: 93. 10.1186/1471-2229-11-93. 21599973 PMC3127826

[fsn372269-bib-0103] Teng, H. , Y. Mi , H. Cao , and L. Chen . 2022. “Enzymatic Acylation of Raspberry Anthocyanin: Evaluations on Its Stability and Oxidative Stress Prevention.” Food Chemistry 372: 130766. 10.1016/j.foodchem.2021.130766. 34600197

[fsn372269-bib-0104] Tuan, P. A. , S. Bai , H. Yaegaki , T. Tamura , S. Hihara , and T. Moriguchi . 2015. “The Crucial Role of *PpMYB10.1* in Anthocyanin Accumulation in Peach and Relationships Between Its Allelic Type and Skin Color Phenotype.” BMC Plant Biology 1, no. 15: 208. 10.1186/s12870-016-0894-8. PMC4652394 26582106

[fsn372269-bib-0105] Verde, I. , A. G. Abbott , S. Scalabrin , et al. 2013. “The High‐Quality Draft Genome of Peach ( *Prunus persica* ) Identifies Unique Patterns of Genetic Diversity, Domestication and Genome Evolution.” Nature Genetics 45, no. 5: 487–494. 10.1038/ng.2586. 23525075

[fsn372269-bib-0106] Wang, H. , H. Zhang , Y. Yang , et al. 2020. “The Control of Red Colour by a Family of MYB Transcription Factors in Octoploid Strawberry (*Fragaria* × *Ananassa*) Fruits.” Plant Biotechnology Journal 18, no. 5: 1169–1184. 10.1111/pbi.13282. 31647169 PMC7152614

[fsn372269-bib-0107] Wang, L. , Y. Gao , L. Zhang , et al. 2025. “Reduced Proteasome Degradation of HsfB2a at Higher Temperatures Is Responsible for the Inhibition of Anthocyanin Synthesis in Pear.” Plant Cell 37, no. 6: koaf102. 10.1093/plcell/koaf102. 40324390

[fsn372269-bib-0108] Wang, L. , Z. Luo , M. Yang , et al. 2022. “The Action of RED Light: Specific Elevation of Pelargonidin‐Based Anthocyanin Through ABA‐Related Pathway in Strawberry.” Postharvest Biology and Technology 186: 111835. 10.1016/j.postharvbio.2022.111835.

[fsn372269-bib-0109] Wang, N. , W. Liu , Z. Mei , et al. 2024. “A Functional InDel in the WRKY10 Promoter Controls the Degree of Flesh Red Pigmentation in Apple.” Advanced Science 11, no. 30: e2400998. 10.1002/advs.202400998. 38874015 PMC11321683

[fsn372269-bib-0110] Wang, S. , Z. Zhang , L. Li , et al. 2022. “Apple MdMYB306‐Like Inhibits Anthocyanin Synthesis by Directly Interacting With MdMYB17 and MdbHLH33.” Plant Journal 110, no. 4: 1021–1034. 10.1111/tpj.15720. 35220614

[fsn372269-bib-0111] Wang, X. , W. Zeng , Y. Ding , et al. 2019. “PpERF3 Positively Regulates ABA Biosynthesis by Activating PpNCED2/3 Transcription During Fruit Ripening in Peach.” Horticulture Research 6: 19. 10.1038/s41438-018-0094-2. 30729009 PMC6355789

[fsn372269-bib-0112] Wang, Y. , Y. Xiao , Y. Sun , et al. 2023. “Two B‐Box Proteins, PavBBX6/9, Positively Regulate Light‐Induced Anthocyanin Accumulation in Sweet Cherry.” Plant Physiology 192, no. 3: 2030–2048. 10.1093/plphys/kiad137. 36930566 PMC10315283

[fsn372269-bib-0113] Xiangzhao, Z. , Z. Lin , X. Xu , Z. Jialing , Q. Shenchun , and W. Sanhong . 2026. “The Influence of Environmental Factors on Anthocyanin Biosynthesis in Fruits.” Plant Science 363: 112866. 10.1016/j.plantsci.2025.112866. 41232723

[fsn372269-bib-0114] Xie, X. , S. Li , R. Zhang , et al. 2012. “The bHLH Transcription Factor MdbHLH3 Promotes Anthocyanin Accumulation and Fruit Colouration in Response to Low Temperature in Apples.” Plant, Cell & Environment 35, no. 11: 1884–1897. 10.1111/j.1365-3040.2012.02523.x. 22519753

[fsn372269-bib-0115] Xing, Y. , W. Sun , Y. Sun , et al. 2023. “MPK6‐Mediated HY5 Phosphorylation Regulates Light‐Induced Anthocyanin Accumulation in Apple Fruit.” Plant Biotechnology Journal 21, no. 2: 283–301. 10.1111/pbi.13941. 36208018 PMC9884024

[fsn372269-bib-0116] Xiong, B. , Y. Li , J. Yao , et al. 2025. “Integration of Transcriptomic and Metabolomic Analysis Reveals Light‐Regulated Anthocyanin Accumulation in the Peel of ‘Yinhongli’ Plum.” BMC Plant Biology 25, no. 1: 391. 10.1186/s12870-025-06414-z. 40148754 PMC11948737

[fsn372269-bib-0117] Xu, H. , N. Wang , J. Liu , et al. 2017. “The Molecular Mechanism Underlying Anthocyanin Metabolism in Apple Using the MdMYB16 and MdbHLH33 Genes.” Plant Molecular Biology 94, no. 1–2: 149–165. 10.1007/s11103-017-0601-0. 28286910

[fsn372269-bib-0118] Xu, R. , Y. Wang , L. Wang , et al. 2023. “PsERF1B‐PsMYB10.1‐PsbHLH3 Module Enhances Anthocyanin Biosynthesis in the Flesh‐Reddening of Amber‐Fleshed Plum (cv. Friar) Fruit in Response to Cold Storage.” Horticulture Research 10, no. 6: uhad91. 10.1093/hr/uhad091. PMC10277908 37342542

[fsn372269-bib-0119] Yan, Y. , J. Chemler , L. Huang , S. Martens , and M. A. G. Koffas . 2005. “Metabolic Engineering of Anthocyanin Biosynthesis in *Escherichia coli* .” Applied and Environmental Microbiology 71, no. 7: 3617–3623. 10.1128/AEM.71.7.3617-3623.2005. 16000769 PMC1169036

[fsn372269-bib-0120] Yanez‐Apam, J. , A. Dominguez‐Uscanga , A. Herrera‐Gonzalez , et al. 2023. “Pharmacological Activities and Chemical Stability of Natural and Enzymatically Acylated Anthocyanins: A Comparative Review.” Pharmaceuticals 16, no. 5: 638. 10.3390/ph16050638. 37242421 PMC10220857

[fsn372269-bib-0121] Yang, T. , H. Ma , J. Zhang , et al. 2019. “Systematic Identification of Long Noncoding RNAs Expressed During Light‐Induced Anthocyanin Accumulation in Apple Fruit.” Plant Journal 100, no. 3: 572–590. 10.1111/tpj.14470. 31344284

[fsn372269-bib-0122] Yang, Y. , G. Zhao , W. Yue , S. Zhang , C. Gu , and J. Wu . 2013. “Molecular Cloning and Gene Expression Differences of the Anthocyanin Biosynthesis‐Related Genes in the Red/Green Skin Color Mutant of Pear ( *Pyrus communis* L.).” Tree Genetics & Genomes 9, no. 5: 1351–1360. 10.1007/s11295-013-0644-6.

[fsn372269-bib-0123] Yao, G. , M. Ming , A. C. Allan , et al. 2017. “Map‐Based Cloning of the Pear Gene MYB114 Identifies an Interaction With Other Transcription Factors to Coordinately Regulate Fruit Anthocyanin Biosynthesis.” Plant Journal 92, no. 3: 437–451. 10.1111/tpj.13666. 28845529

[fsn372269-bib-0124] Yuan, H. , W. Cai , X. Chen , F. Pang , J. Wang , and M. Zhao . 2022. “Heterozygous Frameshift Mutation in FaMYB10 Is Responsible for the Natural Formation of Red and White‐Fleshed Strawberry ( *Fragaria × ananassa* Duch).” Frontiers in Plant Science 13: 1027567. 10.3389/fpls.2022.1027567. 36388497 PMC9644031

[fsn372269-bib-0125] Zhang, S. , H. Wang , T. Wang , et al. 2023. “MdMYB305‐MdbHLH33‐MdMYB10 Regulates Sugar and Anthocyanin Balance in Red‐Fleshed Apple Fruits.” Plant Journal 113, no. 5: 1062–1079. 10.1111/tpj.16100. 36606413

[fsn372269-bib-0126] Zhang, X. , L. Yu , M. Zhang , et al. 2024. “MdWER Interacts With MdERF109 and MdJAZ2 to Mediate Methyl Jasmonate‐ and Light‐Induced Anthocyanin Biosynthesis in Apple Fruit.” Plant Journal 118, no. 5: 1327–1342. 10.1111/tpj.16671. 38319946

[fsn372269-bib-0127] Zhang, Y. , Y. Feng , S. Yang , et al. 2023. “Identification of Flavanone 3‐Hydroxylase Gene Family in Strawberry and Expression Analysis of Fruit at Different Coloring Stages.” International Journal of Molecular Sciences 24, no. 23: 16807. 10.3390/ijms242316807. 38069129 PMC10706444

[fsn372269-bib-0128] Zhang, Z. , Y. Shi , Y. Ma , et al. 2020. “The Strawberry Transcription Factor FaRAV1 Positively Regulates Anthocyanin Accumulation by Activation of FaMYB10 and Anthocyanin Pathway Genes.” Plant Biotechnology Journal 18, no. 11: 2267–2279. 10.1111/pbi.13382. 32216018 PMC7589338

[fsn372269-bib-0129] Zhang, Z. , X. Wang , Y. Gao , et al. 2025. “Orchestrating Anthocyanin Biosynthesis in Fruit of Fruit Trees: Transcriptional, Post‐Transcriptional, and Post‐Translational Regulation.” International Journal of Biological Macromolecules 307, no. 1: 141835. 10.1016/j.ijbiomac.2025.141835. 40064275

[fsn372269-bib-0130] Zhao, J. 2015. “Flavonoid Transport Mechanisms: How to Go, and With Whom.” Trends in Plant Science 20, no. 9: 576–585. 10.1016/j.tplants.2015.06.007. 26205169

[fsn372269-bib-0131] Zhao, T. , Q. Li , T. Yan , B. Yu , Q. Wang , and D. Wang . 2025. “Sugar and Anthocyanins: A Scientific Exploration of Sweet Signals and Natural Pigments.” Plant Science 353: 112409. 10.1016/j.plantsci.2025.112409. 39894058

[fsn372269-bib-0132] Zhao, Y. , C. Wang , X. Huang , and D. Hu . 2021. “Genome‐Wide Analysis of the Glutathione S‐Transferase (GST) Genes and Functional Identification of MdGSTU12 Reveals the Involvement in the Regulation of Anthocyanin Accumulation in Apple.” Genes 12, no. 11: 1733. 10.3390/genes12111733. 34828339 PMC8619396

[fsn372269-bib-0133] Zheng, T. , L. Wei , J. Xiang , J. Wu , and J. Cheng . 2025. “HMGR Modulates Strawberry Fruit Coloration and Aroma Through Regulating Terpenoid and Anthocyanin Pathways.” Food 14, no. 7: 1199. 10.3390/foods14071199. PMC11988353 40238400

[fsn372269-bib-0134] Zhou, H. , K. Lin‐Wang , F. Wang , et al. 2019. “Activator‐Type R2R3‐MYB Genes Induce a Repressor‐Type R2R3‐MYB Gene to Balance Anthocyanin and Proanthocyanidin Accumulation.” New Phytologist 221, no. 4: 1919–1934. 10.1111/nph.15486. 30222199

[fsn372269-bib-0135] Zhou, H. , K. Lin‐Wang , H. Wang , et al. 2015. “Molecular Genetics of Blood‐Fleshed Peach Reveals Activation of Anthocyanin Biosynthesis by NAC Transcription Factors.” Plant Journal 82, no. 1: 105–121. 10.1111/tpj.12792. 25688923

[fsn372269-bib-0136] Zhou, H. , Q. Peng , J. Zhao , et al. 2016. “Multiple R2R3‐MYB Transcription Factors Involved in the Regulation of Anthocyanin Accumulation in Peach Flower.” Frontiers in Plant Science 7: 1557. 10.3389/fpls.2016.01557. 27818667 PMC5073212

[fsn372269-bib-0137] Zhu, Y. , B. Zhang , A. C. Allan , et al. 2020. “DNA Demethylation Is Involved in the Regulation of Temperature‐Dependent Anthocyanin Accumulation in Peach.” Plant Journal 102, no. 5: 965–976. 10.1111/tpj.14680. 31923329

